# Effects of above ground pathogen infection and fungicide application on the root-associated microbiota of apple saplings

**DOI:** 10.1186/s40793-023-00502-z

**Published:** 2023-05-27

**Authors:** Maximilian Fernando Becker, A. Michael Klueken, Claudia Knief

**Affiliations:** 1 Institute of Crop Science and Resource Conservation - Molecular Biology of the Rhizosphere, University of Bonn, Nussallee 13, 53115 Bonn, Germany; 2 Crop Science Division, Disease Control Biology, Bayer AG, Alfred-Nobel-Str. 50, 40789 Monheim am Rhein, Germany

**Keywords:** Rhizosphere, Endosphere, Microbiota, Fungicide, Pathogen infection, Apple

## Abstract

**Background:**

The root-associated microbiome has been of keen research interest especially in the last decade due to the large potential for increasing overall plant performance in agricultural systems. Knowledge about the impact of above ground plant disturbances on the root-associated microbiome remains limited. We addressed this by focusing on two potential impacts, foliar pathogen infection alone and in combination with the application of a plant health protecting product. We hypothesized that these lead to plant-mediated responses in the rhizosphere microbiota.

**Results:**

The effects of an infection of greenhouse grown apple saplings with either *Venturia inaequalis* or *Podosphaera leucotricha* as foliar pathogen, as well as the combined effect of *P. leucotricha* infection and foliar application of the synthetic plant health protecting product Aliette (active ingredient: fosetyl-aluminum), were studied on the root-associated microbiota. The bacterial community structure of rhizospheric soil and endospheric root material was characterized post-infection, using 16S rRNA gene amplicon sequencing. With increasing disease severity both pathogens led to changes in the rhizosphere and endosphere bacterial communities in comparison to uninfected plants (explained variance up to 17.7%). While the preventive application of Aliette on healthy plants two weeks prior inoculation did not induce changes in the root-associated microbiota, a second later application on the diseased plants decreased disease severity and resulted in differences of the rhizosphere bacterial community between infected and several of the cured plants, though differences were overall not statistically significant.

**Conclusions:**

Foliar pathogen infections can induce plant-mediated changes in the root-associated microbiota, indicating that above ground disturbances are reflected in the below-ground microbiome, even though these become evident only upon severe leaf infection. The application of the fungicide Aliette on healthy plants itself did not induce any changes, but the application to diseased plants helped the plant to regain the microbiota of a healthy plant. These findings indicate that above ground agronomic management practices have implications for the root-associated microbiome, which should be considered in the context of microbiome management strategies.

**Supplementary Information:**

The online version contains supplementary material available at 10.1186/s40793-023-00502-z.

## Introduction

The term rhizosphere was first coined in 1904 by the German agronomist and plant physiologist Lorenz Hiltner and is now defined as the narrow region of soil around plant roots, which harbors a specific microbiome with potential benefits for plant health [1-3]. Some rhizosphere microbes have capabilities to enter the root and establish an endophytic lifestyle, thereby undergoing an even closer association and with further possibilities to influence root health and plant growth [4-6]. This root-associated microbiome is mostly recruited from the surrounding soil and is considered to be crucial for healthy agricultural soils and thus for food production (reviewed in [7]). Its composition has been shown to depend on various factors such as the biophysical and biogeochemical environment, but is also actively shaped by the plant [8, 9]. The plant species [10, 11], its genotype [12], spatial heterogeneity within the root system related to root age and differentiation [13, 14] and nutrient acquisition strategies [15] are considered some of the main drivers behind microbiome assembly and dynamics. More specifically, its composition is shaped by the plant via rhizodeposition [16, 17], which itself depends on various factors, including abiotic and biotic influence factors. Abiotic factors include light intensity and temperature [18], the mechanistics of carbon and nitrogen flow into the rhizosphere [19], water supply [20], or the application of plant health protecting products (PHPPs) such as pesticides [21], while biotic factors include the presence of other organisms (reviewed in [22]), and root or even foliar pathogens to which the plant responds [14, 23, 24].

The root-associated microbiome is known to be able to protect plants against stresses such as pathogen infections [25] and has been shown to be actively recruited to suppress soilborne pathogens [26]. Various studies in recent years have shown that different root, above ground or systemic pathogens can impact the root-associated microbiota. Devastating root pathogens such as *Phytophthora* causing root rot [27], above ground fungal pathogens such as *Botrytis cinerea* [28] or *Podosphaera aphanis* on strawberry [29], as well as the phloem-limited bacterial Huanglongbing citrus disease [30] have been shown to induce changes in the microbial rhizosphere community to varying degrees. Beyond this, Gu et al. [31] have recently suggested that small changes in the rhizosphere microbiome can be an early indicator for the presence of a soilborne pathogen. The effects of a pathogen infection can be plant compartment specific as a recent study by Kim et al. [32] shows. Here, the systemic bacterial pathogen *Erwinia amylovora* causing fireblight in several *Rosaceae* was shown to induce significant changes in the apple root endosphere bacterial community composition, whereas the rhizosphere communities remained unchanged. Thus, evidence exists that above ground or systemic pathogens can impact the root-associated microbiota. However, detailed knowledge about this process remains limited; it is in particular unknown how early during the above ground infection process changes become evident in the root-associated microbiota and whether responses are pathogen-specific. Understanding the composition and responses of the root-associated microbial communities of healthy and diseased plants is essential for promoting plant health and growth and has thereby potential to contribute to sustainable agriculture [33, 34].

Currently, disease control relies predominantly on repeated PHPP applications and the integration of non-pesticidal control measures, such as removing litter residues or using resistant plant varieties [35, 36]. The application of synthetic and biological products for disease prevention and reduction is common practice and has recently been found to influence the root-associated microbiota (reviewed in [37]). Depending on the product group and application mode, different effects on the root-associated microbiota have been observed by pesticide applications. PHPPs with direct contact of the compound with the root-associated microbiota have been shown to induce significant effects. For example, soil or seed treatments such as seed coatings, as well as systemic PHPPs have been shown to affect bacterial and fungal rhizosphere communities in maize, soybean, rice, strawberry and sugar cane [38-42]. Similarly, spray applications of the systemic herbicide haloxyfop-R-methyl have been shown to dissipate into the rhizosphere soil upon application and consecutive plant death to influence the soil and rhizosphere bacterial richness and diversity [43]. However, little is known about possible plant mediated effects on the root-associated microbiota when PHPPs are applied above ground. The only currently available study about the effects of spray application of a mixture of the systemic fungicides fosetyl-aluminum and propamocarb-hydrochloride has been shown to have a rather weak and only transient impact on the root-associated microbiota [44]. Thus, effects of PHPPs in direct or close contact with the root-associated microbiome in form of seed/soil treatment or as systemic products have been shown, whereas the effects of above ground product applications and thereby plant mediated responses on the root-associated microbiota are currently largely unknown. However, in commercial fruit tree orchards most pesticides are applied as foliar sprays and less as drench application or seed coating [45]. Moreover, the effects of PHPPs on the root-associated microbiome have been studied on healthy plants, while the combined effects resulting from pathogen infections and PHPP applications remain unknown. This may have additive effects, leading to an even more distinct root-associated microbiota of infected, PHPP treated plants, or may to some extent reduce the infection impact, if plants and their associated microbiota are cured by PHPP treatment.

Aim of this study was to investigate the effects of two above ground fungal pathogens and the interaction with a synthetic fungicide on the root-associated bacterial microbiota. We chose apple (*Malus* × *domestica*) as a model organism due to its economic relevance, its susceptibility to several severe pathogens, and the current disease control methods. It is the third most important fruit in terms of production and consumption worldwide with around 83 Mt of apples produced annually [46, 47]. Sustainable apple production is threatened by both, above and below ground pathogens, causing substantial yield and economic losses. Two of the most important and prominent foliar diseases worldwide are apple scab and powdery mildew caused by the fungi *Venturia inaequalis* and *Podosphaera leucotricha*, respectively [48, 49]. Infections by these pathogens are minimized and plant health is maintained in apple orchards by frequent pesticide applications with high dosages [50].

We hypothesized that (i) foliar pathogen infection changes the apple sapling root-associated bacterial community structure depending on disease severity and pathogen species and (ii) the application of PHPPs promotes the return of the microbiota to that of a healthy plant after a pathogen infection event. To test these hypotheses, we analyzed the root-associated microbiota of young apple plants based on two greenhouse trials. Focus of the first trial (referred to as temporal trial) were pathogen-infection induced changes over time in the root-associated bacterial community of plants, either inoculated with the apple pathogen *V. inaequalis* or *P. leucotricha* in comparison to healthy plants. Focus of the second trial (mixed trial) was to analyze the effect of a *P. leucotricha* infection followed by the application of the synthetic fungicide Aliette (containing fosetyl-aluminum). This locally systemic product was chosen as it is effective against *P. leucotricha* [51] and because it has been shown to only induce weak transient changes in the root-associated microbiota [44]. Its active ingredient, fosetyl-aluminum, is considered to have a low mammalian toxicity and to be rapidly degraded in soil to non-toxic components [52]. In both trials we divided the root-associated microbiota into the “loosely associated” (L-compartment, primarily the rhizosphere) and “tightly associated” (T-compartment, primarily the endosphere) microbiota according to the concept of Donn et al. [53] to study responses compartment-specifically. The bacterial community composition was analyzed by amplicon sequencing of the 16S rRNA marker gene.

## Material and methods

### Soil substrate preparation

For both the temporal and mixed trial, soil in proximity of apple trees was taken from a commercial apple orchard in Buxtehude, Germany. The soil was air dried and sieved through a 2-mm mesh. As growth substrate, 45% of this soil was mixed with 45% sterile silica sand and 10% perlite to improve soil texture and therewith seedling growth. The soil mixture was remoistened two days before use and the watered soil transferred into growing trays or pots.

### Plant cultivation

Commercially available apple seeds (*Malus* x *domestica* Borkh., cv. Pink Lady) were stored for at least two months at − 20 °C for stratification. For use, the seeds were then incubated in sterile distilled water (dH_2_O) for four days at 6 °C. Before the seeds were placed into silica sand for germination, the sand was autoclaved, placed into boxes and wetted with sterile dH_2_O overnight. Germination occurred for two weeks at 4 °C with the lid of the tray closed. Individual seedlings were further cultivated in the prepared soil mixture in growing trays, slightly covered with soil, and left for 17 days with 14-h light phase (> 300 μmol m^−2^ s^−1^, Philips SGR 140, Hamburg, Germany) at 16 °C ± 2 °C and 10-h dark phase at 14 °C ± 2 °C in a glasshouse with 50–70% relative air humidity. Healthy 40-day old seedlings were transferred to 13-cm round pots containing pre-moistured soil mixture together with 0.25 g Basacote 6 M controlled-release fertilizer (Compo Expert, Germany). During the following cultivation period, pest control was conducted by using commercially available beneficial organisms in release sachets (*Neoseiulus cucumeris*, *N. barkeri*, *Phytoseiulus persimilis*, *Amblyseius cucumeris*, *Encarsia formosa*, *Chrysoperla carnea*; Sautter & Stepper, Germany) as biocontrol agents and fungal disease control by weekly sulphur fumigation. To protect the soil from contamination with sulphur, it was covered with felt maps. The pots were drip-irrigated with approximately 10 ml of water per pot per day and their position was randomly changed every week. For both trials, plants were grown under these conditions for five weeks until further treatment.

### Pathogen infection

**Fig. 1:**
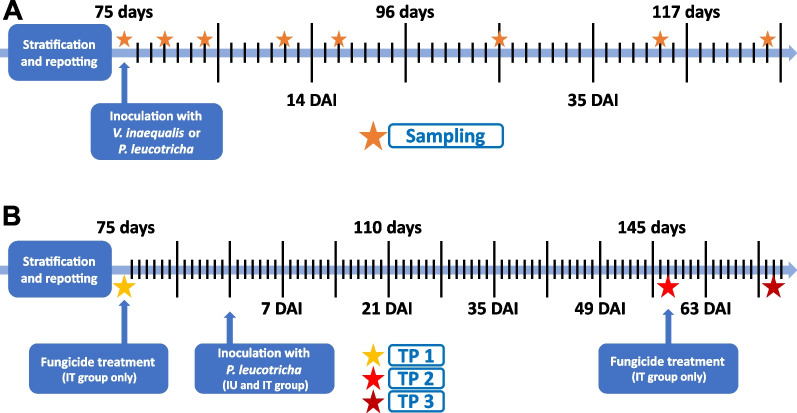
Timeline of the temporal (**A**) and mixed trial (**B**) with inoculation, fungicide application and sampling dates (TP) of apple saplings being indicated. Timelines are labelled with plant age on top and days after infection (DAI) below. The temporal trial consisted of *V. inaequalis* or *P. leucotricha* infected plants and a control treatment without infection. The mixed trial included a treatment with *P. leucotricha* infection without fungicide treatment (inoculated untreated, IU), a treatment with *P. leucotricha* infection and fungicide treatment (inoculated treated, IT), and a non-inoculated untreated control (NC)

Prior pathogen inoculation, plant leaves were rinsed with water to remove sulphur residues. *V. inaequalis* infection of 75-day old apple plants was performed by the method of Steiner and Oerke [54]. Briefly, a *V. inaequalis* spore solution with 10^6^ conidia ml^−1^ was prepared from frozen detached apple leaves (cultivar Pink Lady) with sporulating lesions of strain HS1, which were taken from the strain collection at the INRES Department for Plant Diseases and Plant Protection at the University of Bonn. The spore solution was evenly sprayed onto the plant leaf surfaces. For the untreated control, sterile dH_2_O was sprayed onto the plant surface. Powdery mildew infection was achieved using fresh *P. leucotricha* spores from propagation apple plants (cultivar Pink Lady), as plant material with spores cannot be processed for storage. Diseased apple plants with sporulating colonies on leaves were evenly shaken over the plants. Plants from all three treatments were kept for 48 h in sealable plastic containers filled with roughly 1 cm of water in order to create a humid environment and thus ensure a successful infection with *V. inaequalis* (while maintaining similar conditions between treatments). Additionally, a dark panel was put on top of the boxes for 24 h to decrease light intensity. Afterwards, all plants were placed into climate chambers, whereby the *P. leucotricha* inoculated plants were transferred to a separate chamber to prevent cross-infections. Cross-infection of the control plants by *P. leucotricha* could be excluded because this pathogen requires wet leaf surfaces to germinate. Water supply of the plants was ensured by manually watered mats and both climate chambers were configured to have the same climate conditions (14-h light/10-h dark cycle with 18 °C during daytime and 16 °C during night-time for increased pathogen infection). *V. inaequalis* infected plants were inoculated a second time with the pathogen at 16 days after inoculation (DAI) as described above to achieve an infection of newly developed leaves. To establish the infection, all plants (including *P. leucotricha* treated and control plants without second inoculation) were placed in plastic boxes for 48 h as described above. Samples were taken at different timepoints: 0, 3, 6, 12, 16, 28, 40 and 48 DAI (Fig. 1). At 0 DAI, just before infection, 20 plants were destructively sampled to have a large baseline of untreated plants. Seven to eight replicate plants per treatment were sampled at each timepoint between 3 and 40 DAI with the exception of *P. leucotricha* inoculated plants at 40 DAI. Due to severe disease symptoms all remaining 22 plants were sampled at this timepoint. At 48 DAI, the remaining 15 and 11 replicate plants of the *V. inaequalis* and control group, respectively, were sampled.

In the mixed trial the effects of a *P. leucotricha* infection in combination with a plant health protecting product application on the root-associated microbiota was studied. Therefore, three different treatment groups were sampled at three different timepoints (Fig. 1). The treatment groups consisted of the inoculated treated (IT) group, which received a PHPP treatment, followed by pathogen infection with *P. leucotricha* and a second PHPP treatment after infection; the inoculated untreated (IU) group with just a *P. leucotricha* infection and the non-inoculated untreated control (NC) group without treatments. The IT group received the PHPP treatment prior inoculation to analyze whether a treatment only with a PHPP would cause application effects. At timepoint 1 (TP1), when the apple plants were 75 days old, the systemic product Aliette WG 80H (80% fosetyl-aluminum; 3.0 kg ha^−1^ in 600 l ha^−1^ water) (Bayer AG, Germany) was applied to the IT group, while water was applied to the IU and NC group. After two weeks, plants of the IU and IT group were inoculated with *P. leucotricha* as described above, while water was applied to plants of the NC group. After an additional 58 days, at timepoint 2 (TP2), when disease symptoms were prominent, a second Aliette application was given to the IT group, while water was again applied to the IU and NC group. Twelve replicate samples of the IT and IU group each were taken at TP1 (75-day old plants, 14 days prior inoculation), TP2 (147-day old plants, 58 DAI), and two weeks after TP2 at timepoint 3 (TP3; 161-day old plants, 72 DAI), while the NC group was only sampled at TP3 (Fig. 1).

### Disease documentation and sample collection

In both trials, disease severity (DS) was visually assessed over time on a 0–5 scale (0 = showing no signs of infection, 1 = having a single leaf with a single lesion, 2 = having either two leaves with multiple lesions or multiple leaves having a single lesion, 3 = having at least two leaves with large scale lesions, 4 = having one leaf entirely covered with mycelium, 5 = having multiple leaves entirely covered with mycelium and with leaves close to senescence). Sampling was performed by loosening the soil, carefully pulling out the entire root system and shaking the plant gently until all excess soil was removed. The root system was cut above the root crown and collected in 50-ml falcon tubes. It was immediately stored on ice and frozen at − 80 °C within four hours after sampling. The root samples were further processed to obtain the loosely and tightly root-associated microorganisms as described by Becker et al. [14]. Briefly, excess soil was removed by gently shaking the root, and the root placed in 50-ml falcon tubes with 45 ml of 0.2 mM CaCl_2_ solution. The samples were vortexed 3 × 30 s and left 10 min for sedimentation. The root material (representing the T-compartment) was taken out, freeze dried and ground using a mixer mill. The suspension (representing the L-compartment) was centrifuged, and the pellet freeze dried and homogenized by vortexing.

### DNA extraction, 16S rRNA gene targeted amplicon sequencing and sequence data analysis

Soil and root material underwent DNA extraction and 16S rRNA gene targeted PCR as described by Becker et al. [14]. In brief, DNA was extracted using the NucleoSpin Soil DNA extraction kit (Macherey Nagel, Düren, Germany) and the 16S rRNA gene was amplified using a nested LNA PCR protocol with the primer set 799f-1193r (V5–V7 region). Library preparation and sequencing was performed by Novogene (Cambridge, UK) on a NovaSeq 6000 system (Illumina, San Diego, CA) and generated paired-end reads (2 × 250 bp). All following steps were done separately for the two trials. The raw Illumina sequence reads were processed using a custom bash script with Cutadapt version 3.2 to demultiplex the samples [55]. Primer removal and further processing was done with QIIME2 version 2021.04 [56]. Paired reads were merged with max. 20 allowed differences in the overlapping region for the merging step and max. 1 expected error, quality filtered using the default settings and denoised using deblur with reads trimmed to 350 bp length and a minimum read number of 50 [57]. All further data processing steps were performed similar as described in Becker et al. [14]. Total read numbers after quality filtering, mean number of reads per sample and number of samples remaining after quality filtering are shown in Additional file 1: Table S1.

### Statistical analyses

Statistical analyses were performed within the QIIME2 environment and in R [56, 58]. Disease severity based on a 0–5 rating was analyzed using Kruskal–Wallis non-parametric tests, followed by Dunn’s post-hoc test. Differences in alpha diversity were assessed by richness (ACE) and diversity (Shannon and Inverse Simpson) using a feature table rarefied to 8000 reads per sample. In the temporal trial, generalized least squares models were used with each diversity index as the dependent variable and treatment and timepoint as explanatory variables. The timepoint was used to adjust the temporal autocorrelation. In the mixed trial linear regression was used with the timepoint and treatment grouped and used as explanatory variable (e.g., TP1–IU). This was done because the control was only sampled at the third timepoint. Pairwise comparisons were performed using estimated marginal means in the emmeans package. Differences in the bacterial community composition were determined using the q2-plugin “DEICODE” [59] and visualized either as principle coordinate analysis (PCoA) plot for the temporal trial or as constrained analysis of principle coordinates (CAP) plot for the mixed trial, where the analysis was constrained by the variables treatment, timepoint and disease severity. Statistical differences were calculated using a form of permutational multivariate analysis of variance (PERMANOVA) on the DEICODE distance matrices with 999 permutations, followed by pairwise comparisons with Benjamini–Hochberg correction for multiple testing, resulting in adjusted *p*-values (*p*_adj_) with a strict significance threshold of *p*_adj_ ≤ 0.01. As the order of factors entered into the PERMANOVA formula influences the outcome in an unbalanced design such as ours, the order of factors in the PERMANOVA formula was varied to identify the model explaining variation best. For the temporal trial, the factors treatment, timepoint, disease severity, number of leaves, plant height and relevant interactions were included in the model to identify factors explaining variance. The best fitting models are shown in the results. In addition, analyses of similarities (ANOSIM) were performed to validate PERMANOVA findings. In the mixed trial, the treatment and timepoint variables were grouped and used as explanatory variable, similar as done for alpha diversity analysis. Homogeneity of dispersions between treatments at the individual timepoints was assessed in the temporal trial using permutational analysis of multivariate dispersions (PERMDISP) via the “betadisper” function with 999 permutations. Pairwise differential abundance analysis at phylum and genus level was performed using ANCOM-BC with detection for structural zeros turned off [60]. In both trials, conservative variance estimates of the test statistic were used and *p*-values were adjusted using Holm’s correction with alpha = 0.1.

## Results

### Temporal trial

**Fig. 2:**
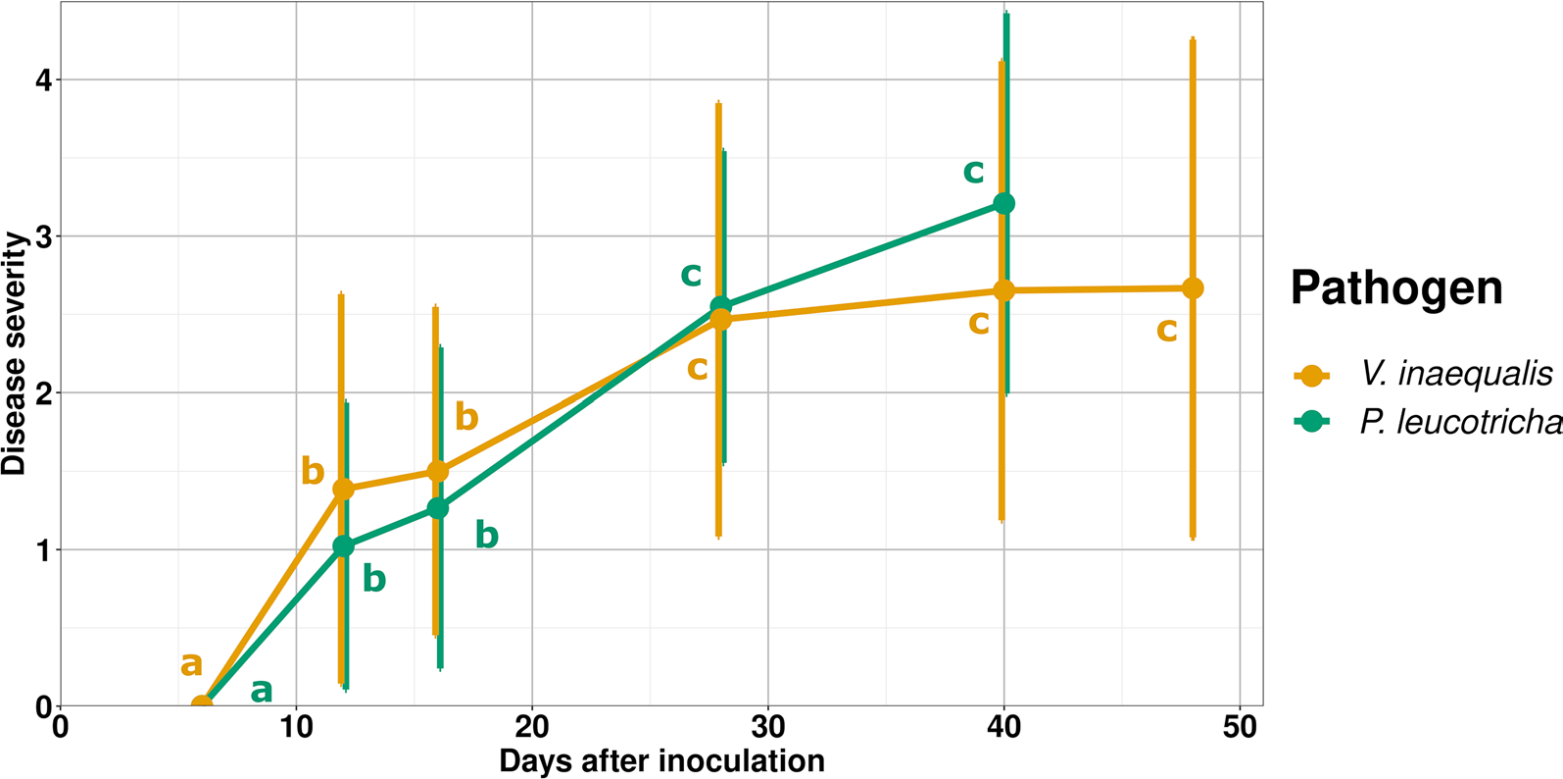
Disease severity of apple saplings infected by *V. inaequalis* or *P. leucotricha*. Disease severity was rated per plant at a 0–5 scale with 0 = healthy and 5 = multiple leaves entirely covered with mycelium and leaves close to senescence. Mean values and standard deviation are displayed based on 11–52 replicates. Significant differences in disease severity were evaluated between different timepoints based on Dunn’s test with Benjamini–Hochberg correction for multiple testing. Lower case letters indicate differences at *p* = 0.05. DAI = days after inoculation

Disease development caused by both pathogens, *V. inaequalis* and *P. leucotricha*, advanced similarly over time. Symptoms became evident twelve days after inoculation (DAI) and disease severity (DS) rose sharply and significantly during the first 28 DAI (Fig. 2). While the mean DS of *V. inaequalis* inoculated plants plateaued afterwards at a level slightly below three, the DS of *P. leucotricha* inoculated plants increased further and caused most plants to wither at 40 DAI, when all remaining plants had to be sampled.

Community compositional analysis showed a dominance of *Proteobacteria* and clear differences between the microbiota of the L- and T-compartment, with *Proteobacteria* being even more pronounced in the T- than the L-compartment (Additional file 1: Fig. S1, ANCOM-BC W = 13.6; *p*_adj_ < 0.001). Likewise, alpha diversity analysis using Shannon’s diversity index showed that the diversity of the bacterial community was significantly lower in the T-compartment (4.6 ± 0.3) compared to the L-compartment (5.9 ± 0.4) (Kruskal–Wallis *p*-value ≤ 0.001). These differences justify a comparative analysis of responses to different pathogen infections over time separately in both fractions. However, the evaluation of treatment effects as well as of temporal changes on the Shannon index applying a generalized least square model revealed no significant differences in either compartment (Table 1). Likewise, richness (ACE) and Inverse Simpson indices remained unaffected by treatments or over time within the compartments.

**Table 1:** Variation in the root-associated bacterial community of apple saplings in dependence on pathogen infection, over time and by other variables. The differences in alpha and beta diversity are summarized for the L- and T-compartment. Effect sizes in beta diversity were assessed by PERMANOVA based on DEICODE distance matrices, while differences in Shannon diversity were analyzed based on generalized least square models (GLS). Significant results (*p* < 0.05) are in bold

Compartment	Variable	PERMANOVA				GLS	
		df	F.Model	R^2^	*p*-value	F.Model	*p*-value
L	Timepoint (TP)	**1**	**62.251**	**0.273**	**0.001**	0.976	0.379
	Treatment	2	1.005	0.009	0.408	0.088	0.768
	Disease severity (DS)	5	0.963	0.021	0.496		
	TP * Treatment	**2**	**4.133**	**0.036**	**0.002**	2.271	0.107
	TP * DS	5	1.22	0.027	0.236		
	Treatment * DS	**6**	**2.349**	**0.062**	**0.002**		
	TP * Treatment * DS	**3**	**2.613**	**0.034**	**0.006**		
T	Timepoint	**1**	**70.770**	**0.291**	**0.001**	0.138	0.242
	Treatment	**2**	**2.268**	**0.019**	**0.048**	2.640	0.075
	Disease severity	5	1.000	0.021	0.441		
	TP * Treatment	**2**	**2.453**	**0.020**	**0.034**	1.649	0.196
	TP * DS	5	0.821	0.017	0.683		
	Treatment * DS	6	0.868	0.021	0.624		
	TP * Treatment * DS	**3**	**2.581**	**0.032**	**0.008**		

**Fig. 3:**
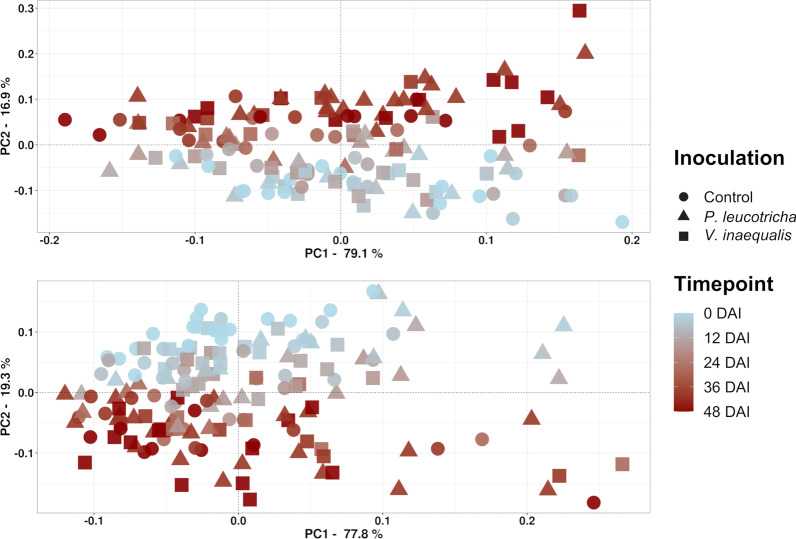
Principle Coordinate Analysis (PCoA) calculated from DEICODE distance matrices, showing variation in the root-associated bacterial community composition of differently inoculated apple saplings over time. Variation in the L-compartment (upper panel) and T-compartment (lower panel) is shown

**Fig. 4:**
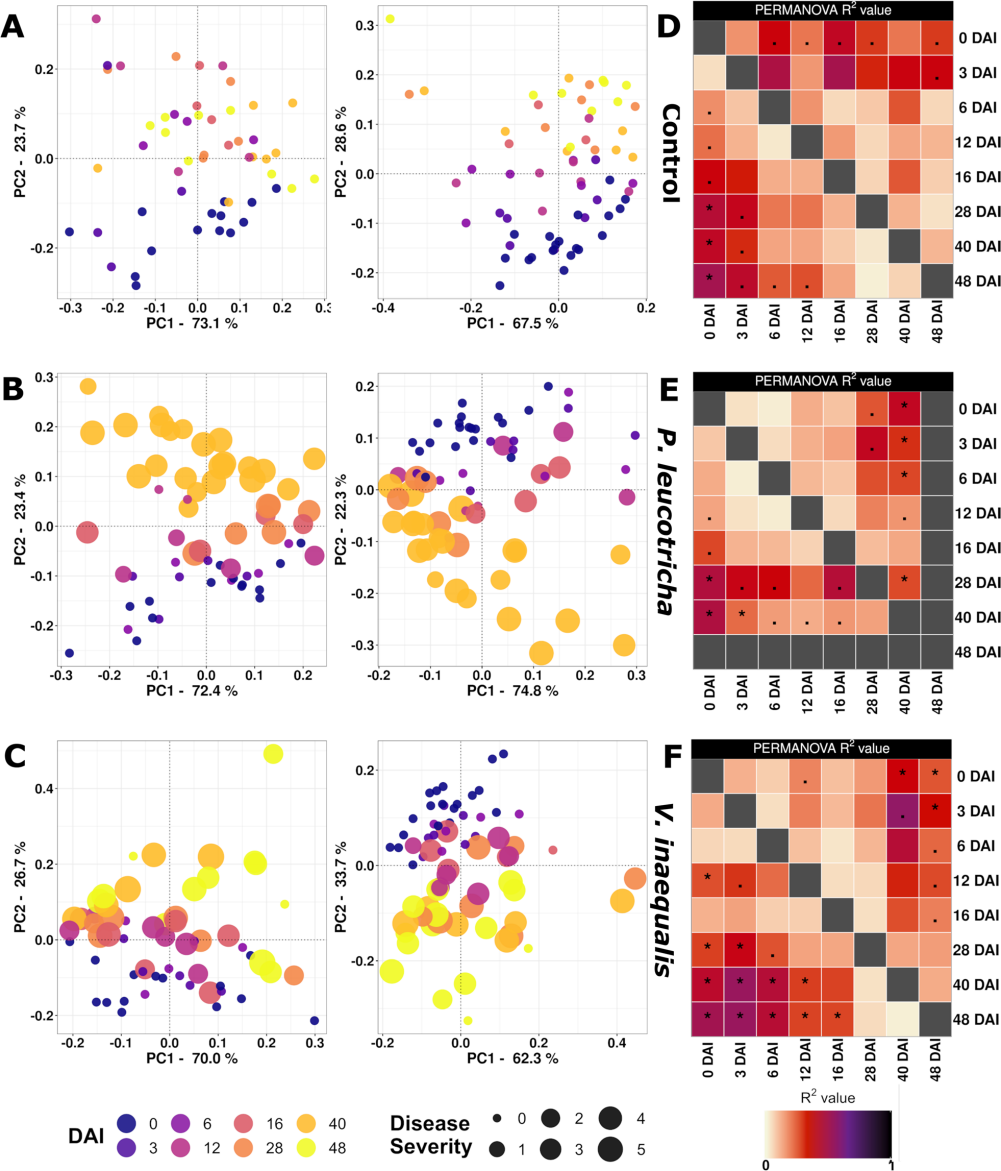
Temporal dynamics in the root-associated bacterial community composition of differently inoculated apple saplings. **A**–**C** Principle Coordinate Analysis (PCoA) based on DEICODE distance matrices, showing variation in the L-compartment (left) and T-compartment (right). **A** Untreated control plants. **B** *P. leucotricha* inoculated plants. **C** *V. inaequalis* inoculated plants. A color code illustrates the different sampling timepoints, point size indicates disease severity based on a 0–5 scale with 0 = plant with healthy leaves and 5 = plant having multiple leaves entirely covered with mycelium and with leaves close to senescence. **D**–**F** PERMANOVA results for pairwise comparisons between timepoints in the three treatment groups: **D** untreated control plants, **E** *P. leucotricha* inoculated plants. **F** *V. inaequalis* inoculated plants. Results for the L-compartment (upper right side) and the T-compartment (lower left side) are shown. R^2^-values are color coded and Benjamini–Hochberg adjusted *p*-values indicated by asterisks, i.e., “*” represents *p* ≤ 0.01 and “.” represents 0.05 ≥ *p* > 0.01. The significance threshold was set at α = 0.01

Regarding beta diversity, we analyzed the relevance of different factors within the PERMANOVA framework. Besides treatment and time, we evaluated plant parameters as measures for plant development and disease severity, thus taking better into account that infected plants developed disease symptoms only at later sampling dates. Because some factors were consequently co-correlated (timepoint, number of leaves and plant height as well as treatment and disease severity), we evaluated different PERMANOVA models comparatively. Both, leaf number and height only explained a negligible part of the variation compared to timepoint and were thus left out in the final PERMANOVA model (Table 1). This was confirmed by ANOSIM, where height and leaf number were identified to be of minor relevance (Additional file 1: Table S2). In the final PERMANOVA model, timepoint was the strongest explanatory factor for bacterial community composition in both compartments (L: R^2^ = 0.273; *p* = 0.001|T: R^2^ = 0.291; *p* = 0.001), whereas the treatment with two different pathogens caused minor differences in the T-compartment (R^2^ = 0.019; *p* = 0.048) and none in the L-compartment (Table 1). The succession over time can be observed in the PCoA plot, where samples are separated along the second axis (Fig. 3), whereas treatment-dependent responses remain invisible. The interaction of timepoint, treatment and DS were significant in both compartments, though only with relatively small R^2^-values of 0.032 and 0.034 (Table 1). As a consequence of those interactive effects, we evaluated the treatment effects at the individual timepoints specifically but found only few significant differences (Additional file 1: Table S3). In the L-compartment, the factor treatment was significant at 40 and 48 DAI (with R^2^ values of 0.144 and 0.102 and *p*-values of 0.038 and 0.043, respectively), whereas the factor DS was significant at 48 DAI, explaining a rather large part of the variation (R^2^ = 0.432; *p* = 0.017). In the T-compartment, PERMANOVA revealed significant differences related to the factor treatment only at 28 DAI (R^2^ = 0.254; *p* = 0.017). Pairwise PERMANOVA however did not result in significant differences at either comparison between the respective treatments and the control. These weak treatment effects, in addition to the combined explanatory power of timepoint * treatment * DS, caused us to also evaluate more closely the temporal dynamics of each treatment separately, where treatment specific differences became more evident (Fig. 4). The community composition of the control group did only significantly change between 0 DAI and the later timepoints at 28, 40 and 48 DAI in the T-compartment with a similar, though insignificant trend in the L-compartment (Fig. 4A, D). In contrast to that, both the community composition of plants inoculated with *P. leucotricha* or *V. inaequalis* shifted differently over time. The community composition of *P. leucotricha* inoculated plants did not change significantly during the first 16 DAI in either compartment, but we observed significant differences at later timepoints, when the disease severity was significantly higher. This was slightly less pronounced at 40 DAI in the T-compartment than in the L-compartment, where the community composition was significantly different to most previous timepoints (Fig. 4E). This shift is also clearly seen in the PCoA plots with samples taken 40 DAI clearly shifting away from the earlier timepoints in both compartments (Fig. 4B). The community composition of *V. inaequalis* inoculated plants displayed differences primarily for samples taken at 40 and 48 DAI compared to samples taken at or prior 16 DAI, especially in the T-compartment (Fig. 4C, F). Thus, they displayed a more similar pattern to plants inoculated with *P. leucotricha* than the non-inoculated control group plants. Again, this trend was also observed in the PCoA plot, where the later timepoints shifted further away from the preceding ones than seen in the control. Besides the differences in the PCoA plot and the PERMANOVA *p*_adj_-values, plants inoculated with *P. leucotricha* or *V. inaequalis* displayed larger R^2^-values and thereby larger differences at the later timepoint comparisons compared to the control, evident from the heatmaps summarizing the PERMANOVA results (Fig. 4D–F).

We performed differential abundance analysis using ANCOM-BC for the three individual treatments between 0 and 40 DAI (Additional file 1: Fig. S2), as in particular the pathogen treated samples shifted significantly away at this timepoint (Fig. 4). Several genera were significantly differentially abundant in one or even both pathogen treatments. However, pathogen-specific enrichments of taxa were not commonly observed, and similar trends were mostly seen in the respective pathogen treatment as well as in the control treatment, suggesting that the pathogen-infection merely enforced the enrichment of these taxa in the rhizosphere.

### Mixed trial

**Fig. 5:**
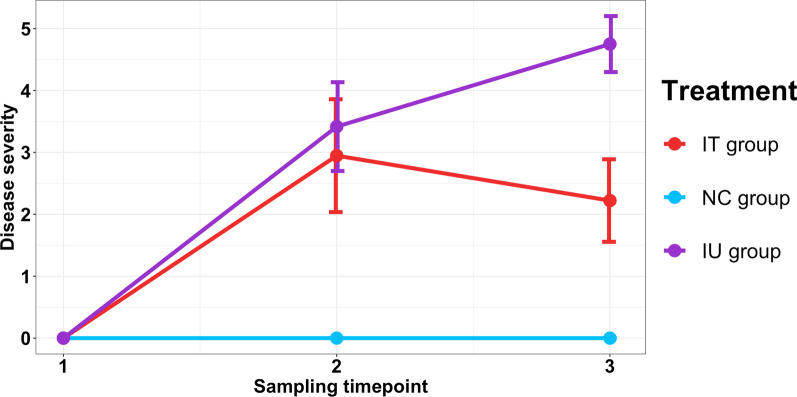
Development of disease severity over three timepoints (TP1–TP3) on *P. leucotricha* infected apple saplings. One inoculated group (IT) was treated with a synthetic fungicide at TP2, the other remained untreated (IU). Both treatments were compared to an uninoculated control group (NC). Disease severity was rated on a 0–5 scale and the mean values and standard deviation of 9–35 replicates are shown. Significant differences in disease severity between the three groups at TP2 and TP3 were assessed by Dunn’s test with Benjamini–Hochberg correction for multiple testing

As *P. leucotricha* showed a stronger disease severity compared to *V. inaequalis* in the first trial, we used *P. leucotricha* in the mixed trial. Plants in both the inoculated untreated (IU) group and inoculated treated (IT) group were similarly infected with *P. leucotricha* and showed lesions at the second timepoint (TP2) 21 days after infection, whereas plants in the non-treated control (NC) group remained healthy (Fig. 5). At TP3, two weeks after the second PHPP application (Fig. 1), the mean disease severity of the IT group decreased slightly to 2.2 ± 0.7, whereas the mean disease severity of the IU group increased significantly to 4.8 ± 0.5, which resulted in significant differences between all three treatment groups (Fig. 5). This decrease was clearly seen as a reduction of the infected leaf area and inhibition of new mycelial growth.

**Fig. 6:**
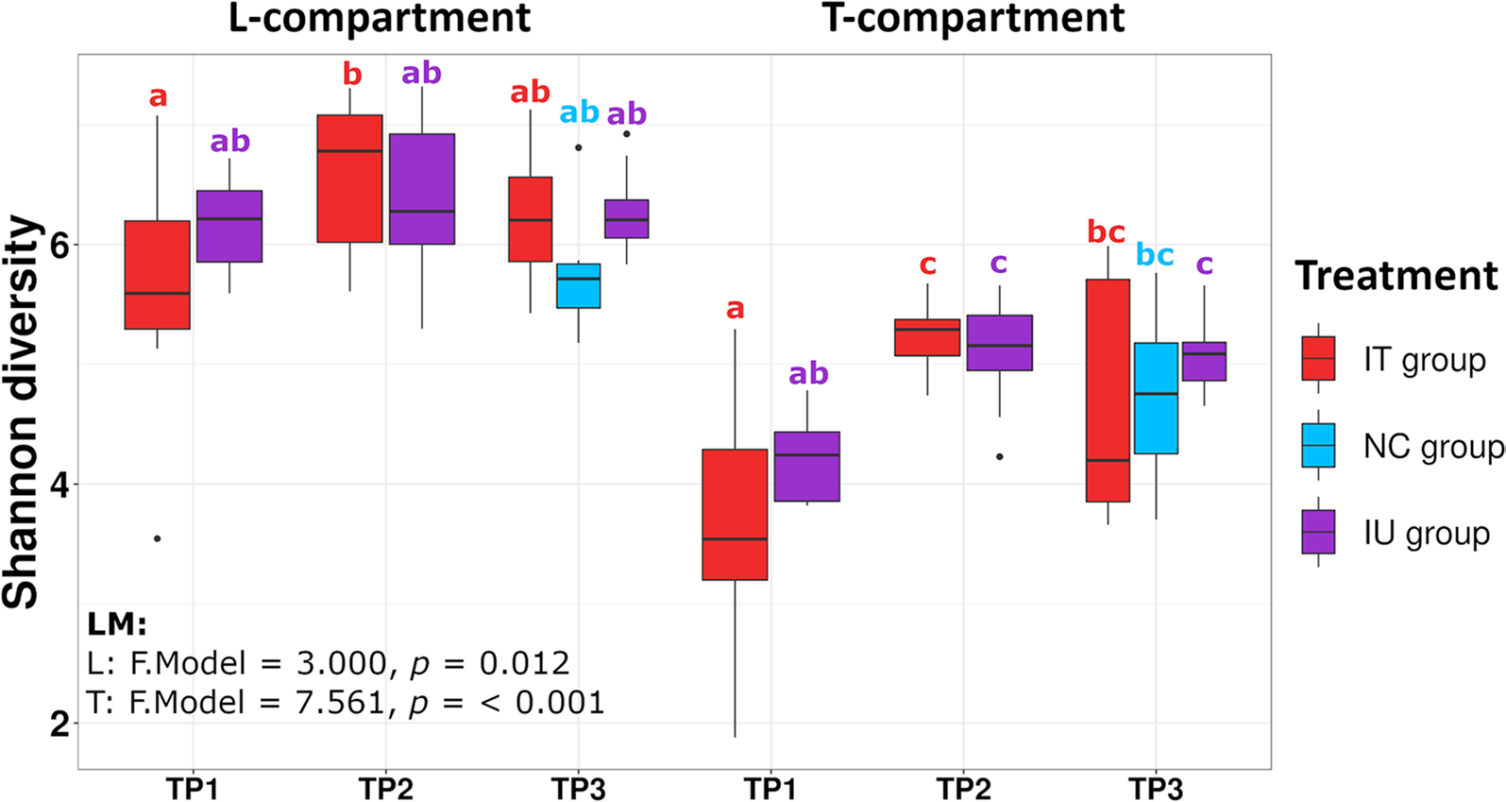
Variation in the root-associated bacterial community of apple saplings linked to treatment and sampling timepoint (TP). The variation in alpha diversity presented based on the Shannon index in the L-compartment (left panel) and T-compartment (right panel). Differences in Shannon diversity were analyzed based on linear models (LM). Different letters represent significant changes according to Tukey’s HSD post hoc tests performed between all seven groups of samples

**Fig. 7:**
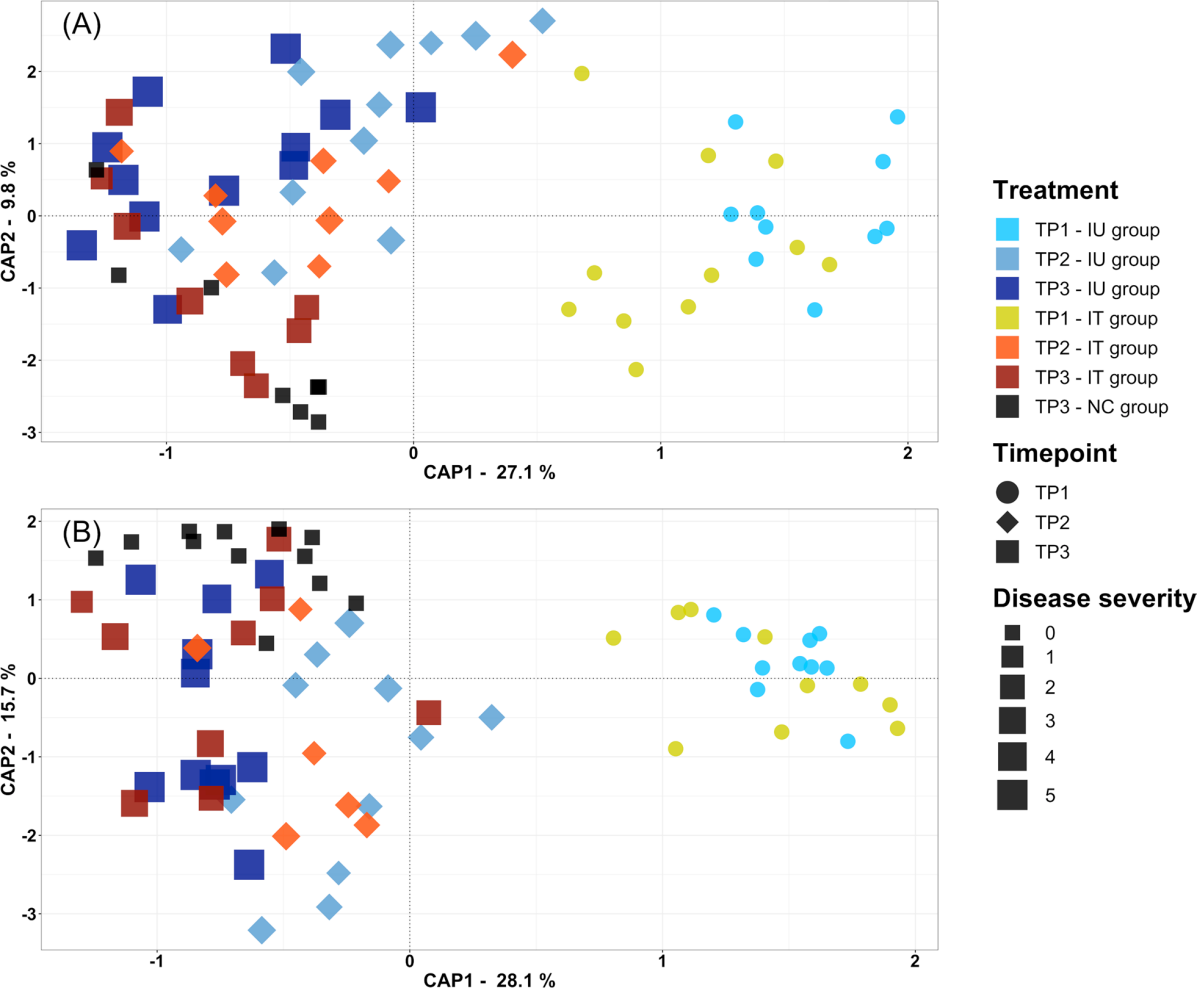
Variation in beta diversity of differently treated apple saplings at three distinct timepoints (TP) in the L-compartment (upper panel) and T-compartment (lower panel). Variation is presented based on constrained analysis of principle coordinates (CAP) using DEICODE distance matrices; it is constrained by the variables sampling timepoint, treatment and disease severity. Plants were either inoculated with *P. leucotricha* and left untreated (IU) or were additionally treated with a synthetic fungicide (IT), or they underwent a treatment with water as control (NC). The different treatments are shown in different colors, and disease severity is illustrated by different symbol sizes, rated on a 0–5 scale with 0 = healthy plants and 5 = plants having multiple leaves entirely covered with mycelium and with leaves close to senescence

**Fig. 8:**
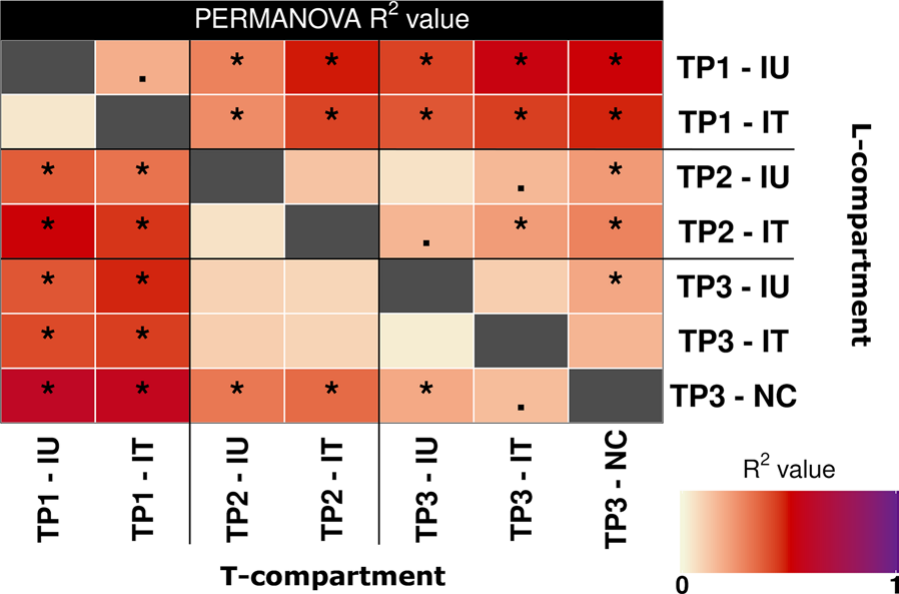
Pairwise PERMANOVA results comparing bacterial community composition in the L- and T-compartment of apple saplings at different timepoints (TP) of three differently treated groups (IU, IT, NC). R^2^-values are illustrated using a color scale with the Benjamini–Hochberg adjusted *p*-values indicated by asterisks, i.e., “*” represents *p* ≤ 0.01 and “.” represents 0.05 ≥ *p* > 0.01. Results for the L-compartment are shown in the upper right side of the figure and for the T-compartment in lower left side. The significance threshold was set at α = 0.01

Similar to the temporal trial, community compositional analysis showed a dominance of *Proteobacteria* and clear differences between the bacterial communities of the L- and T-compartment (Additional file 1: Fig. S3). Because a preliminary CAP and PERMANOVA analysis (not shown) revealed that sampling timepoint was the major explanatory variable and because we had an uneven study design, we combined the variables sampling timepoint and treatment and defined seven categories (e.g., TP1-IU, TP1-IT, TP2-IU) for both alpha and beta diversity analysis. Significant changes in the Shannon diversity index related to the grouped variables were seen in both compartments according to linear models (L: *p* = 0.012|T: *p* < 0.001) (Fig. 6). Focusing on differences between treatments, diversity tended to be higher in the inoculated groups in the L-compartment compared to the control, but subsequent post-hoc tests revealed no significant differences between the treatments at the individual timepoints (Fig. 6). For variation in beta diversity, PERMANOVA and CAP revealed significant differences in the bacterial community composition in both compartments by the grouped variables (Tables 2 and 3, Fig. 7 and Additional file 1: Fig. S4). Subsetting the data by time to assess the treatment effects in more detail (Table 3) as well as displaying all pairwise comparisons in a heatmap (Fig. 8) revealed that the different treatments were significantly different only at TP3. Here, the IU group differed significantly from the NC group in both compartments (L: R^2^ = 0.195; *p*_adj_ = 0.004|T: R^2^ = 0.192; *p*_adj_ = 0.003) (Fig. 8). The IT group however did not differ significantly from the NC group in either compartment, which can also be seen in the CAP plot of the L-compartment, where the IT samples were more similar to those of the NC group than to those of the IT group. This was particularly true for samples with a lower DS within the IT group, which can be seen when comparing plants from TP2 to TP3 in the CAP plot (Fig. 7). While the community composition of the IU group did not change significantly in the two weeks from TP2 to TP3 in either compartment, the community of the IT group samples changed significantly in the L-compartment over time (R^2^ = 0.223; *p*_adj_ = 0.009), likely caused by samples with a smaller DS. The same trend was observed in the T-compartment; however, here the differences were not significant. The differences between treatments at TP3 were also clearly visible in the L-compartment when analyzing TP3 separately in CAP plots (Additional file 1: Fig. S4). Likewise, the DEICODE distances of the two treatments to the control and the within control distances reflected this (Additional file 1: Fig. S5). No differences were found between the IT group and the NC group, but the IU group differed significantly from both the IT and NC group in the L-compartment. This trend persisted for DEICODE distances in the T-compartment; however here, the IT group differed significantly from the NC group and not the IU group.

**Table 2:** Variation in the root-associated bacterial community of apple saplings linked to treatment and sampling timepoint (TP). Differences in beta diversity are first shown related to the combined variables treatment and timepoint (Grouped) in the L- and T-compartment. Below, treatment effects at the individual timepoints are listed. Effect sizes were assessed by PERMANOVA based on DEICODE distance matrices. Significant results (*p* < 0.05) are in bold

Compartment	Variable		PERMANOVA			
			df	F.Model	*R*^2^	*p*-value
L	Grouped		**6**	**10.044**	**0.493**	**0.001**
	TP 1	Treatment	1	1.681	0.081	0.157
	TP 2	Treatment	1	1.355	0.070	0.240
	TP 3	Treatment	**2**	**2.763**	**0.181**	**0.016**
T	Grouped		**6**	**10.085**	**0.490**	**0.001**
	TP 1	Treatment	1	1.485	0.076	0.202
	TP 2	Treatment	1	1.147	0.071	0.327
	TP 3	Treatment	**2**	**3.238**	**0.178**	**0.010**

**Table 3:** Variation in the root-associated bacterial community of apple saplings linked to sampling timepoint (TP). Pairwise differences in beta diversity between individual timepoints in the L- and T-compartment are displayed. Effect sizes were assessed by PERMANOVA based on DEICODE distance matrices. Significant results (*p* < 0.05) are in bold

Compartment	Variable	PERMANOVA		
		F.Model	R^2^	*p*-value
L	TP1–TP2	**15.896**	**0.290**	**0.001**
	TP2–TP3	**4.327**	**0.086**	**0.005**
	TP1–TP3	**26.054**	**0.357**	**0.001**
T	TP1–TP2	**21.646**	**0.382**	**0.001**
	TP2–TP3	**6.323**	**0.116**	**0.002**
	TP1–TP3	**32.377**	**0.388**	**0.001**

**Fig. 9:**
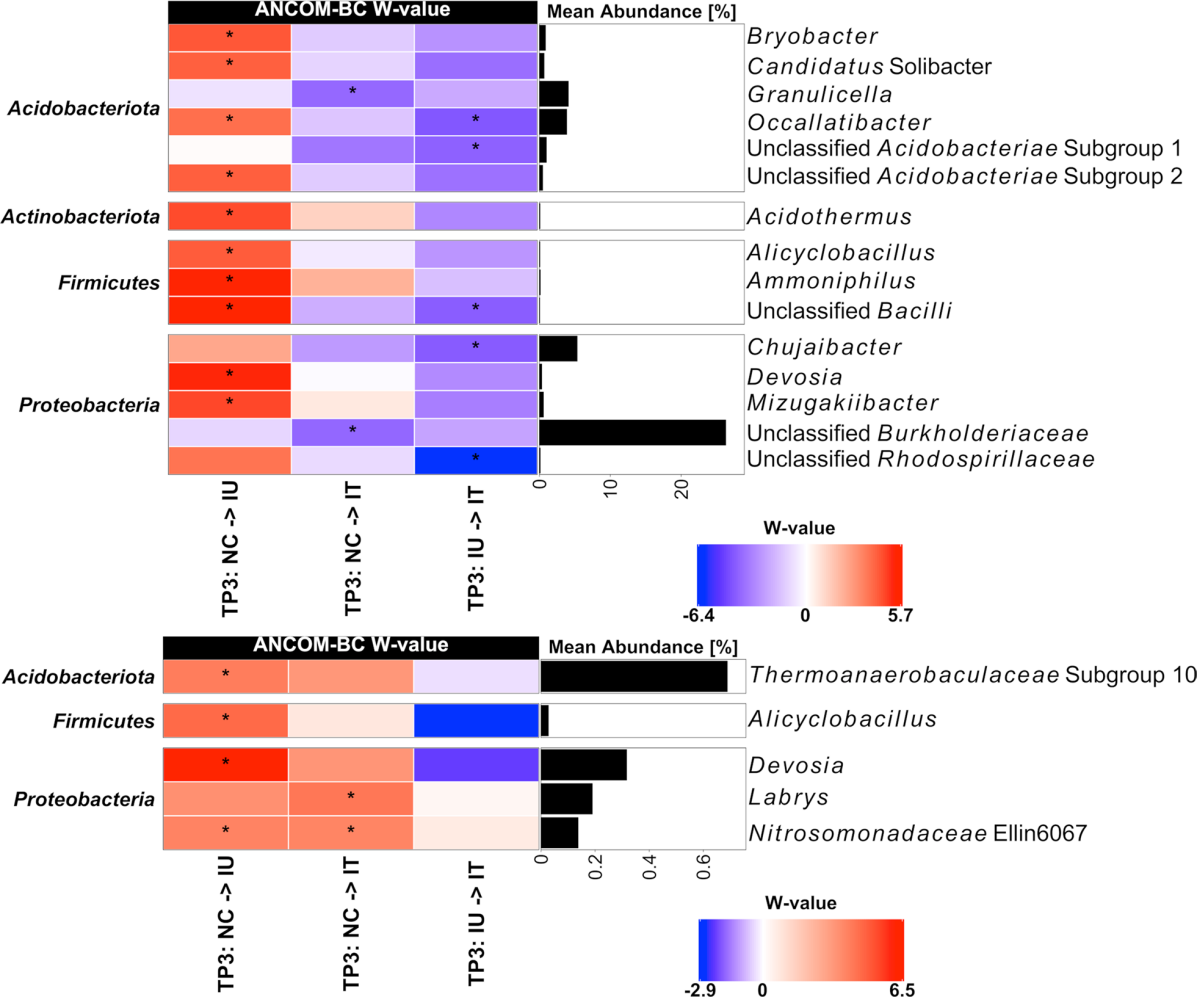
Differential abundance analysis performed at genus level by ANCOM-BC of differently treated apple saplings at the last sampling timepoint (TP3). Results are shown for the L- and T-associated bacterial communities (upper and lower panel, respectively). Plants were inoculated with *P. leucotricha* and then left untreated (IU) or treated with a synthetic fungicide (IT) or remained uninoculated and treated with water as control (NC). The heatmap shows the coefficients obtained from the ANCOM-BC log-linear model divided by their standard error (called W-value). The colour code indicates differential abundances between two treatments with red indicating enrichment in the last value of the column name. A “*” is shown if ANCOM-BC showed significant differences based on adjusted *p*-values in this comparison. In addition, the mean relative abundances of the taxa are displayed and only taxa with a mean relative abundance of > 0.1% are shown

Considering the differences observed between treatments at TP3, a differential abundance analysis was performed based on ANCOM-BC to identify bacterial genera responding to the treatments at this last timepoint (Fig. 9). In both compartments, most responsive taxa were identified between the IU and NC treatment, and additionally, IT appeared less different to NC than IU in the L-compartment. In the IU group, we observed exclusively taxa with significant increases in relative abundance compared to the NC group. In the L-compartment, several *Acidobacteriota* (*Bryobacter*, “*Candidatus* Solibacter*”*, *Ocallatibacter*, an unclassified member of subgroup 2 in the *Acidobacteriae*), several *Firmicutes* (*Alicyclobacillus*, *Ammoniphilus*, an unclassified taxon of the *Bacilli*) and *Proteobacteria* (*Devosia* and *Mizugakiibacter*) showed significant increases in relative abundance. Among these, *Devosia* and *Allicyclobacillus* were also enriched in the T-compartment, besides two further genera.

## Discussion

### Above ground pathogen infection causes changes in the root-associated bacterial community structure

In two separate experimental trials, we analysed potential foliar pathogen infection induced changes over time in the root-associated bacterial community of apple plants. First with a focus on temporal dynamics, then by assessing the combined effect of pathogen infection and plant health product application. In both trials, we divided the root-associated microbiota into the L-compartment (primarily rhizosphere colonizers) and T-compartment (primarily endosphere colonizers).

Based on previous studies [28-30], we expected to observe pathogen related changes in the root-associated bacterial community. However, changes were not necessarily consistent in the literature. In this study, only small differences in alpha diversity caused by pathogen inoculation were seen. No significant changes in Shannon diversity were observed in the temporal trial, while diversity in the two pathogen treated groups of the mixed trial tended to be slightly higher in the inoculated groups compared to the untreated control group in the L-compartment (Fig. 6). In comparison, existing studies showed no differences in alpha diversity upon above [61] or below ground pathogen infection [32], whereas one with powdery mildew in strawberry showed higher Shannon index values in the rhizosphere of healthy plants [29], and another one with *B. cinerea* leaf infection in strawberry showed a higher richness in the rhizosphere of diseased plants [28], similar to our results. A higher diversity has been hypothesized to act as an insurance for maintaining plant productivity under changing environmental conditions [62], which was possibly also seen here under disease stress, though primarily in the rhizosphere and not in the endophytic fraction. Altered rhizodeposition, a main driver of rhizosphere assembly selection, might have been the cause for this increase in diversity.

Regarding beta diversity, we observed a rather weak response to pathogen infections, whereas temporal dynamics over the observational period turned out to be more pronounced (Tables 1 and 2, Figs. 3 and 7). Especially in the temporal trial, the temporal dynamics contributed interactively with treatment-related responses to observed differences and thus hint towards differences in the bacterial community composition (Table 1). Direct comparisons between the two pathogen treatments with the untreated control at the individual timepoints did not result in significant differences, but the succession over time was treatment dependent. The bacterial communities of the pathogen infected plants became most distinct at the later sampling times compared to earlier timepoints and thus with increasing disease severity, while the communities of healthy plants were most distinct at the earliest timepoint (Fig. 4). The different temporal dynamics at later timepoints of diseased plants are probably related to disease progression, whereas healthy plants developed and maintained a balanced and more stable bacterial community over time. This is supported by our findings in the mixed trial, where the differences between the inoculated untreated (IU) group and the negative control also hinted to increasing pathogen related effects on the bacterial community composition over time (Fig. 8). Thus, the effects of an above ground pathogen infection on the root-associated microbiota are likely to increase over time as disease severity increases, especially in the L-compartment. This partially confirms our hypothesis that the root-associated bacterial community is affected by above ground pathogen infection, even though the responses remained weaker than expected.

Several studies have shown a severe impact of root pathogen infections on the rhizobacterial community composition, including changes in density, diversity and functioning. When studying systemic bacterial pathogens, one study reported that the phloem-limited bacterial Huanglongbing citrus disease caused a shift of the rhizosphere bacterial community composition towards a bulk soil-like community [30], whereas another with *Erwinia amylovora*, a systemic bacterial pathogen causing fireblight, only induced changes in the endosphere, not the rhizosphere [32]. Our study suggests that similar processes may occur when plants are infected by pathogens that cause disease symptoms only locally above ground. This is in line with the findings of Yang et al. [29], who found that a powdery mildew infection of strawberry influenced the richness of prokaryotic and fungal communities in rhizosphere soil slightly as well as the relative abundance of several taxa. The larger effects of root pathogens or systemically infecting pathogens appears likely, caused by the more intimate relation between the pathogens and the root-associated microbiota as part of the plant holobiont. Thus, the ability of the plant to alter its associated microbiota and even more so to recruit a beneficial microbiota probably depends on the kind of pathogen and the level of infection.

We performed differential abundance analysis to examine if specific taxa are recruited upon pathogen infection. This revealed several taxa that increased in relative abundance at timepoint 3 in the IU group compared to the healthy control group, especially in the L-compartment. Almost all identified taxa have been shown to profit from different organic carbon compounds in the rhizosphere and are thus likely to respond to alterations in rhizodeposition. *Bryobacter* and “*Candidatus* Solibacter*”* have been shown to be closely related to soil carbon metabolism, as they are sensitive to labile carbon and can be influenced by the addition of straw into the soil [63-66]. *Ammoniphilus*, *Mizugakiibacter*, *Acidothermus* and *Alicyclobacillus* have also been shown to utilize various plant-derived carbon compounds such as glucose, cellulose or oxalacetate [65, 67-69]. Lastly, *Devosia* is positively correlated with soil organic carbon compounds [70, 71]. The increase in relative abundance of all these taxa indicates that the above ground pathogen infection may lead to alterations in root exudation, which then leads to changes in the rhizosphere microbial community composition. Altered root exudation upon foliar infection has already been shown in vitro for *B. cinerea* on tomato and cucumber plants, which then lead to an increase of the chemoattractive effect on the beneficial soil microbe *Trichoderma harzianum* [72]. In our study, the increased relative abundance of the cellulolytic taxon *Acidothermus* in the L-compartment at high disease severity levels could further indicate that microbes might begin to actively hydrolyze root tissue, as the disease-stressed plant may have less capabilities to defend itself. However, the roots did not yet display symptoms of decay. The potential underlying rhizodeposition processes resulting in the observed changes in the rhizosphere microbiota deserve further studies.

Besides a mere response of the rhizosphere microbiota to altered rhizodeposition, it has been shown that plants can selectively recruit beneficial bacteria such as *Bacillus subtilis* as a “cry for help” mechanism against pathogen attack by producing specific chemical compounds and releasing them into the rhizosphere [73-77]. The “cry for help” mechanism has so far primarily been suggested for plant infections with root pathogens such as *Phytophthora* [27], *Ralstonia solanacearum* [78] or *Fusarium pseudograminearum* [26]. Additionally, a downy mildew infection in *Arabidopsis* leaves led to the promotion of a beneficial bacterial consortium in the rhizosphere [79]. In this study, we found two genera known to include strains with plant beneficial properties being increased in relative abundance in the diseased group, an unclassified member of *Bacilli*, as well as *Devosia* [71, 80]. This points to an extension of the concept to above ground infection and deserves more attention in the future.

It is remarkable how long the plants in our study maintained their preferred bacterial community even after showing clear signs of infection above ground. This is in contrast to the claim that alterations in the rhizosphere microbiota can serve as early indicator for pathogen infection [31], at least for above ground pathogens. In contrast, it is in accordance with a recent study, in which authors reported that wheat plants are capable of selecting its preferred root-associated microbiota even under stress conditions and recruit microbes with potential antagonistic activities [81]. In our study, the community compositional analysis of the untreated control groups in both trials revealed high relative abundances of *Granulicella*, *Chujaibacter*, *Burkholderia-Caballeronia-Paraburkholderia*, *Acidipila*, *Bryocella*, *Occallatibacter* and unclassified members of *Burkholderiaceae* and *Acidobacteriaceae* Subgroup 1 in the L-compartment (data not shown). Several *Granulicella* species have been shown to have plant growth promoting abilities [82] and *Chujaibacter* has been associated with nitrogen cycling reactions [83], thus making them typical rhizosphere colonizers. In the T-compartment some of the most prominent taxa in both trials such as *Streptomyces* or members of the family of *Comamonadaceae* have been shown to be associated root endophytes of apple before [14, 84]. Thus, the soil used in this study provided a soil microbial reservoir from which the apple saplings could recruit microbes with potential benefits and taxa well-known to colonize the apple rhizosphere, as we would have expected.

### Fungicide application reverts pathogen induced bacterial community compositional shifts

Aliette is a systemic PHPP with fast degradation in the soil and has likely only a limited and transient impact on the soil bacterial community composition [44]. Whereas it is primarily registered for use against oomycetes, it has been shown to decrease the disease severity of *P. leucotricha* in apples and pears [51]. Even though it is a locally systemic PHPP and can be applied as protective treatment, we did not observe a protective effect in comparison to the untreated control plants when it was applied two weeks prior to the massive pathogen inoculation that followed (Fig. 5). However, a second PHPP application after plant infection at 58 DAI decreased the disease severity of the IT group significantly in comparison to the IU group until TP3, demonstrating its curative properties. Its mode of action is still not fully understood, but it acts by inhibiting the germination process of fungal spores and the development of mycelium upon contact [85]. Besides this direct effect, an indirect mode of action involving the promotion of plant defenses is believed to be involved.

When comparing the community composition between the different treatments, we did not see any changes in the bacterial community composition upon the first PHPP application (Table 2). However, it was striking to observe that the composition of the IT group became in part similar to the NC group again after the second PHPP application at TP3, especially in the L-compartment, whereas the community of the IU plants did not show this development (Figs. 7, 8, and 9; Additional file 1: Figs. S4 and S5). Furthermore, the IT group changed significantly from TP2 to TP3, whereas the IU group did not, indicating that this change was triggered by the application of Aliette at TP2 (Fig. 8). We consider this to be a response of the bacterial community to plant-dependent processes rather than a direct effect of Aliette, even though its active ingredient, fosetyl-aluminum, has been shown to impact the soil microbiota [44]. This was primarily observed for the soil protist community, but not for bacterial community composition. Alpha diversity was only weakly and very transiently affected and the complexity of a bacterial co-occurrence network was only slightly decreased in that study. As we aimed to prevent a direct contact between the fungicide and the soil microbiome by covering the soil surface with felt maps, direct effects of Aliette on the rhizosphere bacterial community were unlikely to occur in this work. Thus, as hypothesized, the observed return of the rhizosphere bacterial community from a diseased plant to that of a healthy plant was likely due to the plant regaining its ability to recruit its “healthy” root-associated microbiota with decreasing disease severity. Our observation that this change occurred apparently faster in the rhizosphere than in the endosphere could mean that the plant is quicker to readjust its microbiome in the rhizosphere by the process of rhizodeposition than in the endosphere after this kind of disturbance.

## Conclusions

Our study demonstrates that the root-associated bacterial community of apple saplings is sensitive to plant-mediated effects resulting from above ground pathogen infections. With increasing disease severity, the two foliar pathogens *V. inaequalis* and *P. leucotricha* induced a continuous shift away from a bacterial community composition of a healthy plant. However, changes were rather subtle and without clear evidence for highly pathogen-specific responses. Genera associated with the conversion of various organic carbon compounds became enriched in the L-compartment of diseased plants with increasing disease severity, indicating that rhizodepositional processes may have changed, thereby leading to the alterations in the rhizosphere microbiota. Compared to studies with root pathogens, these disease related effects resulting from leaf pathogens on the bacterial community structure appear to be weaker and were only visible after longer inoculation periods with higher disease severity. Disease related effects on the rhizosphere microbiota appear thus to depend on both, the kind of pathogen and the disease severity. The responses to above ground pathogen infection are also likely compartment specific, as they were first noted in the tightly associated microbiota but were more pronounced at later timepoints in the loosely associated microbiota. Further, our results suggest that the curative effect of our applied PHPP fosetyl-aluminum on the root-associated microbiome is due to the plant regaining its ability to reestablish the microbiome of a healthy plant. This is apparently achieved faster in the loosely associated microbiota than in the endophytic counterpart. Based on our findings, we conclude that the impacts of pathogen infection and PHPP application on the root-associated microbiome need to be considered when developing microbiome management strategies in the context of sustainable agriculture.

## Supplementary Information

**Additional file 1. Table S1.** The hierarchies in each of the trials with the number of samples. Total read number after quality filtering, mean number of reads per sample and the number of samples remaining after quality filtering. **Table S2.** Differences in beta diversity of the root-associated bacterial community of apple saplings in the L- and T-compartment inoculated with two different pathogens and sampled at different timepoints. The disease severity, the height of the plant and the number of leaves was measured at each timepoint. Effect sizes were assessed by ANOSIM based on DEICODE distance matrices. Significant resultsare printed bold. **Table S3.** Differences in beta diversity of the root-associated bacterial community of apple saplings in the L- and T-compartment treated with two different pathogens and sampled at different timepoints. Effect sizes were assessed by PERMANOVA based on DEICODE distance matrices. Significant resultsare printed bold. **Table S4.** Dispersion of the root-associated bacterial community of young apple plants between different treatments at different timepoints in the L- and T-compartment. The different treatments included inoculations with either *V. inaequalis*or *P. leucotricha* and an uninoculated control. Effect sizes were assessed by PERMDISP and Tukey’s test based on DEICODE distance matrices and p-values adjusted after multiple comparison. Significant results are printed bold. **Figure S1.** Composition of the root-associated bacterial community of apple plants as revealed by 16S rRNA gene amplicon sequencing in the temporal trial. The relative abundance of bacterial families in samples from three different treatments sampled at different days after inoculation in the loosely associated and tightly associated compartment is shown. Phyla and their families with < 2% relative abundance in the respective treatment were grouped as “Other”. **Figure S2.** Differential abundance analysis of genera in the L- and T-compartment in dependence on pathogen infection 40 days after inoculation compared to 0 DAI based on ANCOM-BC. Plants were either inoculated with *V. inaequalis*or *P. leucotricha* and are shown besides an uninoculated control treatment. The heatmap shows the coefficients obtained from the ANCOM-BC log-linear model divided by their standard error. The colour code indicates differential abundances of genera between the two timepoints with red indicating an increase in relative abundance at 40 DAI compared to 0 DAI. A “*” is shown if ANCOM-BC showed significant differences using the adjusted p-value in this comparison. The mean relative abundances of the taxa are displayed at 40 DAI in percent. In the L‑compartment, most identified genera of the pathogen inoculated plants belong to the phylum *Proteobacteria*, e.g., unclassified members of the *Comamonadaceae*, *Moraxellaceae*, *Morganellaceae*, *Sphingomonadaceae* and *Xanthomonadaceae*. In the control plants, several *Acidobacteriota* such as *Acidipila*, *Bryobacter* or *Bryocella* were significantly decreased in relative abundance at 40 DAI, though not in the inoculated plants. Only few observations like these were made in the T-compartment with *Edaphobacter*, *Acidibacter* and unclassified members of *Methylophilaceae* and *Micropepsaceae* being significantly increased in the inoculated plants 40 DAI, but not in the control plants. **Figure S3.** Composition of the root-associated bacterial community of apple plants as revealed by 16S rRNA gene amplicon sequencing in the mixed trial. The relative abundance of bacterial families in samples from three different treatments at three different timepoints in the loosely associated and tightly associated compartment is shown. Phyla and their families with < 2% relative abundance in the respective treatment were grouped as “Other”. **Figure S4.** Variation in beta diversity of differently treated apple saplings at timepoint 3 in the L-compartment and T-compartment. Variation is presented based on constrained analysis of principle coordinates using DEICODE distance matrices; it is constrained by the variables treatment and disease severity. Plants were either inoculated with *P. leucotricha* and left untreated or were additionally treated with a synthetic fungicide, or they underwent a treatment with water as control. The different treatments are shown in different colors, and disease severity is illustrated by different symbol sizes, rated on a 0-5 scale with 0 = healthy plants and 5 = plants having multiple leaves entirely covered with mycelium and with leaves close to senescence. **Figure S5.** Boxplots showing the differences between two different treatments to an untreated control group for the L-compartment and T-compartment at TP3 based on DEICODE distances. Significant differences were calculated with pairwise Kruskal-Wallis tests.

## Data Availability

The datasets supporting the conclusions of this article are available in the Sequence Read Archive (SRA) repository under BioProject ID PRJNA899679 (https://www.ncbi.nlm.nih.gov/bioproject/PRJNA899679). A full record of all statistical analyses in R, the metadata, the unrarefied ASV tables and the corresponding taxonomic classifications is available in GitHub (https://github.com/mfbeuq/becker_etal_abovegroundpathogen).

## Abbreviations

ASV: Amplicon sequence variant
CAP: Constrained analysis of principal coordinates
DAI: Days after inoculation
DS: Disease severity
IT: Inoculated treated
IU: Inoculated untreated
L: Loosely associated
NC: Uninoculated untreated control
*p*_adj_: Adjusted p-values
PCoA: Principal coordinate analysis
PHPP: Plant health protecting product
T: Tightly associated

## References

[1] Hiltner L (1904). Über neuere Erfahrungen und Probleme auf dem Gebiete der Bodenbakteriologie unter besonderer Berücksichtigung der Gründüngung und Brache. Arb Dtsch Landwirtsch Ges.

[2] Ali MA, Naveed M, Mustafa A, Abbas A, Kumar V (2017). The good, the bad, and the ugly of rhizosphere microbiome. Probiotics and plant health.

[3] Mendes R, Garbeva P, Raaijmakers JM (2013). The rhizosphere microbiome: significance of plant beneficial, plant pathogenic, and human pathogenic microorganisms. FEMS Microbiol Rev.

[4] Frank AC, Saldierna Guzmán JP, Shay JE (2017). Transmission of bacterial endophytes. Microorganisms.

[5] Araujo R, Dunlap C, Barnett S, Franco CMM (2019). Decoding wheat endosphere-rhizosphere microbiomes in *Rhizoctonia solani*-infested soils challenged by *Streptomyces* biocontrol agents. Front Plant Sci.

[6] White JF, Kingsley KL, Zhang Q, Verma R, Obi N, Dvinskikh S (2019). Review: endophytic microbes and their potential applications in crop management. Pest Manag Sci.

[7] Vries FT, Wallenstein MD (2017). Below-ground connections underlying above-ground food production: a framework for optimising ecological connections in the rhizosphere. J Ecol.

[8] Costa R, Götz M, Mrotzek N, Lottmann J, Berg G, Smalla K (2006). Effects of site and plant species on rhizosphere community structure as revealed by molecular analysis of microbial guilds. FEMS Microbiol Ecol.

[9] de la Fuente Cantó C, Simonin M, King E, Moulin L, Bennett MJ, Castrillo G, Laplaze L (2020). An extended root phenotype: the rhizosphere, its formation and impacts on plant fitness. Plant J.

[10] Garbeva P, van Elsas JD, van Veen JA (2008). Rhizosphere microbial community and its response to plant species and soil history. Plant Soil.

[11] Berg G, Smalla K (2009). Plant species and soil type cooperatively shape the structure and function of microbial communities in the rhizosphere. FEMS Microbiol Ecol.

[12] Micallef SA, Shiaris MP, Colón-Carmona A (2009). Influence of *Arabidopsis thaliana* accessions on rhizobacterial communities and natural variation in root exudates. J Exp Bot.

[13] Bonkowski M, Tarkka M, Razavi BS, Schmidt H, Blagodatskaya E, Koller R (2021). Spatiotemporal dynamics of maize (*Zea mays* L.) root growth and its potential consequences for the assembly of the rhizosphere microbiota. Front Microbiol.

[14] Becker MF, Hellmann M, Knief C (2022). Spatio-temporal variation in the root-associated microbiota of orchard-grown apple trees. Environ Microbiomes.

[15] Guyonnet JP, Guillemet M, Dubost A, Simon L, Ortet P, Barakat M (2018). Plant nutrient resource use strategies shape active rhizosphere microbiota through root exudation. Front Plant Sci.

[16] Haichar FEZ, Marol C, Berge O, Rangel-Castro JI, Prosser JI, Balesdent J (2008). Plant host habitat and root exudates shape soil bacterial community structure. ISME J.

[17] Zhalnina K, Louie KB, Hao Z, Mansoori N, Da Rocha UN, Shi S (2018). Dynamic root exudate chemistry and microbial substrate preferences drive patterns in rhizosphere microbial community assembly. Nat Microbiol.

[18] Pramanik MHR, Nagai M, Asao T, Matsui Y (2000). Effects of temperature and photoperiod on phytotoxic root exudates of cucumber (*Cucumis sativus*) in hydroponic culture. J Chem Ecol.

[19] Jones DL, Nguyen C, Finlay RD (2009). Carbon flow in the rhizosphere: carbon trading at the soil–root interface. Plant Soil.

[20] Henry A, Doucette W, Norton J, Bugbee B (2007). Changes in crested wheatgrass root exudation caused by flood, drought, and nutrient stress. J Environ Qual.

[21] Dinelli G, Bonetti A, Marotti I, Minelli M, Busi S, Catizone P (2007). Root exudation of diclofop-methyl and triasulfuron from foliar-treated durum wheat and ryegrass. Weed Res.

[22] Dessaux Y, Grandclément C, Faure D (2016). Engineering the rhizosphere. Trends Plant Sci.

[23] Yuan J, Zhao J, Wen T, Zhao M, Li R, Goossens P (2018). Root exudates drive the soil-borne legacy of aboveground pathogen infection. Microbiome.

[24] Wen T, Zhao M, Yuan J, Kowalchuk GA, Shen Q (2021). Root exudates mediate plant defense against foliar pathogens by recruiting beneficial microbes. Soil Ecol Lett.

[25] Finkel OM, Castrillo G, Paredes SH, González IS, Dangl JL (2017). Understanding and exploiting plant beneficial microbes. Curr Opin Plant Biol.

[26] Liu H, Li J, Carvalhais LC, Percy CD, Prakash Verma J, Schenk PM, Singh BK (2021). Evidence for the plant recruitment of beneficial microbes to suppress soil-borne pathogens. New Phytol.

[27] Solís-García IA, Ceballos-Luna O, Cortazar-Murillo EM, Desgarennes D, Garay-Serrano E, Patiño-Conde V (2020). *Phytophthora* root rot modifies the composition of the avocado rhizosphere microbiome and increases the abundance of opportunistic fungal pathogens. Front Microbiol.

[28] de Tender C, Haegeman A, Vandecasteele B, Clement L, Cremelie P, Dawyndt P (2016). Dynamics in the strawberry rhizosphere microbiome in response to biochar and *Botrytis cinerea* leaf infection. Front Microbiol.

[29] Yang J, Wei S, Su D, Zhang Z, Chen S, Luo Z (2020). Comparison of the rhizosphere soil microbial community structure and diversity between powdery mildew-infected and noninfected strawberry plants in a greenhouse by high-throughput sequencing technology. Curr Microbiol.

[30] Trivedi P, He Z, van Nostrand JD, Albrigo G, Zhou J, Wang N (2012). Huanglongbing alters the structure and functional diversity of microbial communities associated with citrus rhizosphere. ISME J.

[31] Gu Y, Banerjee S, Dini-Andreote F, Xu Y, Shen Q, Jousset A, Wei Z (2022). Small changes in rhizosphere microbiome composition predict disease outcomes earlier than pathogen density variations. ISME J.

[32] Kim SH, Cho G, Lee SI, Kim DR, Kwak YS (2021). Comparison of bacterial community of healthy and *Erwinia amylovora* infected apples. Plant Pathol J.

[33] Lynch JM, Leij F de. Rhizosphere. e LS. 2001.

[34] Busby PE, Soman C, Wagner MR, Friesen ML, Kremer J, Bennett A (2017). Research priorities for harnessing plant microbiomes in sustainable agriculture. PLoS Biol.

[35] Carisse O, Bernier J (2002). Effect of environmental factors on growth, pycnidial production and spore germination of *Microsphaeropsis* isolates with biocontrol potential against apple scab. Mycol Res.

[36] Michalecka M, Masny S, Leroy T, Puławska J (2018). Population structure of *Venturia inaequalis*, a causal agent of apple scab, in response to heterogeneous apple tree cultivation. BMC Evol Biol.

[37] Ramakrishnan B, Maddela NR, Venkateswarlu K, Megharaj M (2021). Linkages between plant rhizosphere and animal gut environments: interaction effects of pesticides with their microbiomes. Environ Adv.

[38] Huang W, Lu Y, Chen L, Sun D, An Y (2021). Impact of pesticide/fertilizer mixtures on the rhizosphere microbial community of field-grown sugarcane. 3 Biotech.

[39] Nettles R, Watkins J, Ricks K, Boyer M, Licht M, Atwood LW (2016). Influence of pesticide seed treatments on rhizosphere fungal and bacterial communities and leaf fungal endophyte communities in maize and soybean. Appl Soil Ecol.

[40] Kusstatscher P, Wicaksono WA, Thenappan DP, Adam E, Müller H, Berg G (2020). Microbiome management by biological and chemical treatments in maize is linked to plant health. Microorganisms.

[41] Deng S, Wipf HM-L, Pierroz G, Raab TK, Khanna R, Coleman-Derr D (2019). A plant growth-promoting microbial soil amendment dynamically alters the strawberry root bacterial microbiome. Sci Rep.

[42] Qian H, Zhu Y, Chen S, Jin Y, Lavoie M, Ke M, Fu Z (2018). Interacting effect of diclofop-methyl on the rice rhizosphere microbiome and denitrification. Pestic Biochem Physiol.

[43] Liang Q, Yan Z, Li X (2020). Influence of the herbicide haloxyfop-R-methyl on bacterial diversity in rhizosphere soil of *Spartina alterniflora*. Ecotoxicol Environ Saf.

[44] Fournier B, Dos Santos SP, Gustavsen JA, Imfeld G, Lamy F, Mitchell EAD (2020). Impact of a synthetic fungicide (fosetyl-Al and propamocarb-hydrochloride) and a biopesticide (*Clonostachys rosea*) on soil bacterial, fungal, and protist communities. Sci Total Environ.

[45] Thompson A, Holton R, KC A, Moretti M, Wiman N. 2023 Pest management guide for tree fruits. 2023. https://catalog.extension.oregonstate.edu/sites/catalog/files/project/pdf/em8203.pdf. Accessed 31 Mar 2023.

[46] Khajuria YP, Kaul S, Wani AA, Dhar MK (2018). Genetics of resistance in apple against *Venturia inaequalis* (Wint.) Cke. Tree Genet Genomes.

[47] Ritchie H, Rosado P, Roser M. Agricultural production. 2020. https://ourworldindata.org/agricultural-production. Accessed 25 Jan 2022.

[48] Bowen JK, Mesarich CH, Bus VGM, Beresford RM, Plummer KM, Templeton MD (2011). *Venturia inaequalis*: the causal agent of apple scab. Mol Plant Pathol.

[49] Tian X, Zhang L, Feng S, Zhao Z, Wang X, Gao H (2019). Transcriptome analysis of apple leaves in response to powdery mildew (*Podosphaera leucotricha*) infection. Int J Mol Sci.

[50] Eurostat. The use of plant protection products in the European Union: Data 1992–2003. 2007.

[51] Petré R, Labourdette G, Braun CA, Meredith R, Hauke K, van Hemelrijck W (2015). Fosetyl-Al (Aliette^®^), a plant defense enhancer with good efficacy on bacteria and on ascomycetes in apples and pears. Acta Hortic.

[52] Lewis KA, Tzilivakis J, Warner DJ, Green A (2016). An international database for pesticide risk assessments and management. Hum Ecol Risk Assess Int J.

[53] Donn S, Kirkegaard JA, Perera G, Richardson AE, Watt M (2015). Evolution of bacterial communities in the wheat crop rhizosphere. Environ Microbiol.

[54] Steiner U, Oerke E-C (2007). Localized melanization of appressoria is required for pathogenicity of *Venturia inaequalis*. Phytopathology.

[55] Martin M. Cutadapt removes adapter sequences from high-throughput sequencing reads. EMBnet J. 2011;17(1). 10.14806/ej.17.1.200

[56] Bolyen E, Rideout JR, Dillon MR, Bokulich NA, Abnet CC, Al-Ghalith GA (2019). Reproducible, interactive, scalable and extensible microbiome data science using QIIME 2. Nat Biotechnol.

[57] Amir A, McDonald D, Navas-Molina JA, Kopylova E, Morton JT, Zech Xu Z, Kightley EP, Thompson LR, Hyde ER, Gonzalez A, Knight R. Deblur rapidly resolves single-nucleotide community sequence patterns. ABSTRACT mSystems. 2017;2(2). 10.1128/mSystems.00191-16 10.1128/mSystems.00191-16 PMC5340863 28289731

[58] R Core Team. R: a language and environment for statistical computing. Vienna, Austria; 2021.

[59] Martino C, Morton JT, Marotz CA, Thompson LR, Tripathi A, Knight R, Zengler K (2019). A novel sparse compositional technique reveals microbial perturbations. mSystems.

[60] Lin H, Peddada SD (2020). Analysis of compositions of microbiomes with bias correction. Nat Commun.

[61] González-Escobedo R, Muñoz-Castellanos LN, Muñoz-Ramirez ZY, López CG, Avila-Quezada GD. Microbial community analysis of rhizosphere of healthy and wilted pepper (*Capsicum annuum L.*) in an organic farming system: Research Square Platform LLC; 2021.

[62] Wagg C, Jansa J, Schmid B, van der Heijden MGA (2011). Belowground biodiversity effects of plant symbionts support aboveground productivity. Ecol Lett.

[63] Yu Z, Li Y, Wang G, Liu J, Liu J, Liu X (2016). Effectiveness of elevated CO_2_ mediating bacterial communities in the soybean rhizosphere depends on genotypes. Agric Ecosyst Environ.

[64] Wang YH, Yu ZH, Li YS, Wang GH, Tang C, Liu XB (2019). Elevated CO_2_ alters the structure of the bacterial community assimilating plant-derived carbon in the rhizosphere of soya bean. Eur J Soil Sci.

[65] Li R, Liu J, Li J, Sun C (2020). Straw input can parallelly influence the bacterial and chemical characteristics of maize rhizosphere. Environ Pollut Bioavailab.

[66] Zhou W, Qin X, Lyu D, Qin S (2021). Effect of glucose on the soil bacterial diversity and function in the rhizosphere of *Cerasus sachalinensis*. Hortic Plant J.

[67] Sahin N (2003). Oxalotrophic bacteria. Res Microbiol.

[68] Talia P, Sede SM, Campos E, Rorig M, Principi D, Tosto D (2012). Biodiversity characterization of cellulolytic bacteria present on native Chaco soil by comparison of ribosomal RNA genes. Res Microbiol.

[69] Liao H, Li Y, Yao H (2019). Biochar amendment stimulates utilization of plant-derived carbon by soil bacteria in an intercropping system. Front Microbiol.

[70] Chen S, Waghmode TR, Sun R, Kuramae EE, Hu C, Liu B (2019). Root-associated microbiomes of wheat under the combined effect of plant development and nitrogen fertilization. Microbiome.

[71] Chhetri G, Kim I, Kang M, Kim J, So Y, Seo T (2022). *Devosia rhizoryzae* sp. Nov., and *Devosia oryziradicis* sp. nov., novel plant growth promoting members of the genus *Devosia*, isolated from the rhizosphere of rice plants. J Microbiol.

[72] Lombardi N, Vitale S, Turrà D, Reverberi M, Fanelli C, Vinale F (2018). Root exudates of stressed plants stimulate and attract *Trichoderma s*oil fungi. Mol Plant-Microbe Interact.

[73] Rudrappa T, Czymmek KJ, Paré PW, Bais HP (2008). Root-secreted malic acid recruits beneficial soil bacteria. Plant Physiol.

[74] Jousset A, Rochat L, Lanoue A, Bonkowski M, Keel C, Scheu S (2011). Plants respond to pathogen infection by enhancing the antifungal gene expression of root-associated bacteria. Mol Plant-Microbe Interact.

[75] Jousset A, Becker J, Chatterjee S, Karlovsky P, Scheu S, Eisenhauer N (2014). Biodiversity and species identity shape the antifungal activity of bacterial communities. Ecology.

[76] Dudenhöffer J-H, Scheu S, Jousset A (2016). Systemic enrichment of antifungal traits in the rhizosphere microbiome after pathogen attack. J Ecol.

[77] Schulz-Bohm K, Gerards S, Hundscheid M, Melenhorst J, de Boer W, Garbeva P (2018). Calling from distance: attraction of soil bacteria by plant root volatiles. ISME J.

[78] Wei Z, Hu J, Gu Y, Yin S, Xu Y, Jousset A (2018). *Ralstonia solanacearum* pathogen disrupts bacterial rhizosphere microbiome during an invasion. Soil Biol Biochem.

[79] Berendsen RL, Vismans G, Yu K, Song Y, de Jonge R, Burgman WP (2018). Disease-induced assemblage of a plant-beneficial bacterial consortium. ISME J.

[80] Akinrinlola RJ, Yuen GY, Drijber RA, Adesemoye AO (2018). Evaluation of *Bacillus s*trains for plant growth promotion and predictability of efficacy by in-vitro physiological traits. Int J Microbiol.

[81] Yin C, Casa Vargas JM, Schlatter DC, Hagerty CH, Hulbert SH, Paulitz TC (2021). Rhizosphere community selection reveals bacteria associated with reduced root disease. Microbiome.

[82] Kielak AM, Cipriano MAP, Kuramae EE (2016). Acidobacteria strains from subdivision 1 act as plant growth-promoting bacteria. Arch Microbiol.

[83] Cloutier M, Chatterjee D, Elango D, Cui J, Bruns MA, Chopra S (2021). Sorghum root flavonoid chemistry, cultivar, and frost stress effects on rhizosphere bacteria and fungi. Phytobiomes J.

[84] Mahnkopp-Dirks F, Radl V, Kublik S, Gschwendtner S, Schloter M, Winkelmann T (2020). Molecular barcoding reveals the genus *Streptomyces* as associated root endophytes of apple (*Malus domestica*) plants grown in soils affected by apple replant disease. Phytobiomes J.

[85] Krämer W, editor. Modern crop protection compounds. 2nd edn. Weinheim: Chichester; 2012.

