# Supplementary material for: Effects of above ground pathogen infection and fungicide application on the root-associated microbiota of apple saplings
Source: Environ Microbiome. 2023 May 27;18:43. doi: 10.1186/s40793-023-00502-z (PMC10225105; doi:10.1186/s40793-023-00502-z)
Supplement: Supplementary file 1 — Additional file 1. Table S1. The hierarchies in each of the trials with the number of samples. Total read number after quality filtering, mean number of reads per sample and the number of samples remaining after quality filtering. Table S2. Differences in beta diversity of the root-associated bacterial community of apple saplings in the L- and T-compartment inoculated with two different pathogens and sampled at different timepoints. The disease severity, the height of the plant and the number of leaves was measured at each timepoint. Effect sizes were assessed by ANOSIM based on DEICODE distance matrices. Significant resultsare printed bold. Table S3. Differences in beta diversity of the root-associated bacterial community of apple saplings in the L- and T-compartment treated with two different pathogens and sampled at different timepoints. Effect sizes were assessed by PERMANOVA based on DEICODE distance matrices. Significant resultsare printed bold. Table S4. Dispersion of the root-associated bacterial community of young apple plants between different treatments at different timepoints in the L- and T-compartment. The different treatments included inoculations with either V. inaequalisor P. leucotricha and an uninoculated control. Effect sizes were assessed by PERMDISP and Tukey’s test based on DEICODE distance matrices and p-values adjusted after multiple comparison. Significant results are printed bold. Figure S1. Composition of the root-associated bacterial community of apple plants as revealed by 16S rRNA gene amplicon sequencing in the temporal trial. The relative abundance of bacterial families in samples from three different treatments sampled at different days after inoculation in the loosely associated and tightly associated compartment is shown. Phyla and their families with < 2% relative abundance in the respective treatment were grouped as “Other”. Figure S2. Differential abundance analysis of genera in the L- and T-compartment in dependence on pathoge [file 40793_2023_502_MOESM1_ESM.pdf]

Supplemental

Maximilian Becker, A. Michael Klueken, Claudia Knief

Effects of above ground pathogen infection and fungicide application on the root-associated microbiota of apple saplings

Table S1. The hierarchies in each of the trials with the number of samples. Total read number after quality filtering, mean number of reads per sample and the number of samples remaining after quality filtering.

Trial	Hierarchical structure	Number of samples	Total number of reads	Mean number of reads/sample	Samples remaining after quality filtering
Temporal trial	(⌀8 plants x 7 timepoints x 3 treatments + 20 baseline control plants) x 2 compartments	376 root associated samples	18.193.861	58.501	311 samples
Mixed trial	(12 plants x 3 timepoints x 2 treatments + 12 untreated control plants) x 2 compartments	168 root associated samples	4.516.967	32.496	139 samples

Table S2. Differences in beta diversity of the root-associated bacterial community of apple saplings in the L- and T-compartment inoculated with two different pathogens (*V. inaequalis* or *P.leucotricha*, additionally a negative control) and sampled at different timepoints. The disease severity, the height of the plant and the number of leaves was measured at each timepoint. Effect sizes were assessed by ANOSIM based on DEICODE distance matrices. Significant results (p < 0.05) are in bold.

**Factor**	**L-compartment**		**T-compartment**	
	**R**	***p*-value**	**R**	***p*-value**
Treatment	0.007	0.209	**0.035**	**0.010**
Timepoint	**0.350**	**0.001**	**0.334**	**0.001**
Disease severity	**0.112**	**0.001**	**0.092**	**0.005**
Height	**0.109**	**0.027**	0.074	0.080
Number of leaves	0.020	0.128	**0.050**	**0.007**

Table S3. Differences in beta diversity of the root-associated bacterial community of apple saplings in the L- and T-compartment treated with two different pathogens and sampled at different timepoints (TP). Effect sizes were assessed by PERMANOVA based on DEICODE distance matrices. Significant results ($p < 0.05$) are in bold.

TP	Factor	L-compartment				T-compartment			
		**df**	**R^2**	**F**	***p*-value**	**df**	**R^2**	**F**	***p*-value**
**3**	Treatment	2	0.105	0.703	0.647	2	0.129	1.335	0.260
**6**	Treatment	2	0.022	0.114	0.975	2	0.119	0.882	0.509
**12**	Treatment	2	0.153	1.345	0.282	2	0.096	1.060	0.380
	DS	3	0.223	1.307	0.308	3	0.267	1.971	0.104
	Treatment * DS	1	0.057	1.009	0.375	1	0.050	1.101	0.386
**16**	Treatment	2	0.148	1.196	0.357	2	0.122	0.692	0.654
	DS	2	0.263	2.122	0.151	2	0.084	0.479	0.759
	Treatment * DS	1	0.091	1.474	0.241	1	0.267	1.517	0.271
**28**	Treatment	2	0.246	2.110	0.086	**2**	**0.254**	**2.998**	**0.017**
	DS	3	0.062	0.356	0.850	3	0.128	1.006	0.428
	Treatment * DS	1	0.052	0.891	0.419	1	0.151	3.552	0.099
**40**	Treatment	**2**	**0.144**	**2.721**	**0.038**	2	0.094	1.576	0.187
	DS	4	0.138	1.305	0.255	4	0.071	0.595	0.738
	Treatment * DS	1	0.033	1.239	0.310	1	0.062	1.047	0.313
**48**	Treatment	**1**	**0.102**	**3.268**	**0.043**	1	0.002	0.036	0.973
	DS	**4**	**0.432**	**3.467**	**0.017**	4	0.071	0.459	0.830

Table S4. Dispersion of the root-associated bacterial community of young apple plants between different treatments at different timepoints (DAI) in the L- and T-compartment. The different treatments included inoculations with either *V. inaequalis* (V) or *P. leucotricha* (L) and a uninoculated control (C). Effect sizes were assessed by PERMDISP and Tukey's test based on DEICODE distance matrices and *p*-values adjusted after multiple comparison. Significant results are in bold.

DAI	Comparison	L-compartment		T-compartment	
		Difference	p_{adj}	Difference	p_{adj}
3	L-C	-0.408	0.794	0.850	0.133
	V-C	-0.877	0.343	-0.360	0.671
	V-L	-0.469	0.691	**-1.210**	**0.025**
6	L-C	-0.049	0.997	0.704	0.222
	V-C	0.164	0.971	-0.334	0.689
	V-L	0.213	0.947	-1.039	0.066
12	L-C	0.096	0.985	0.924	0.223
	V-C	-0.451	0.726	0.059	0.993
	V-L	-0.547	0.600	-0.865	0.265
16	L-C	0.520	0.780	0.607	0.577
	V-C	0.267	0.923	-0.296	0.872
	V-L	-0.253	0.931	-0.903	0.230
28	L-C	-0.682	0.350	-0.405	0.796
	V-C	-0.180	0.925	0.139	0.968
	V-L	0.503	0.555	0.544	0.648
40	L-C	0.199	0.812	0.204	0.889
	V-C	-0.250	0.834	0.291	0.865
	V-L	-0.449	0.451	0.087	0.981
48	V-C	0.473	0.147	0.131	0.762

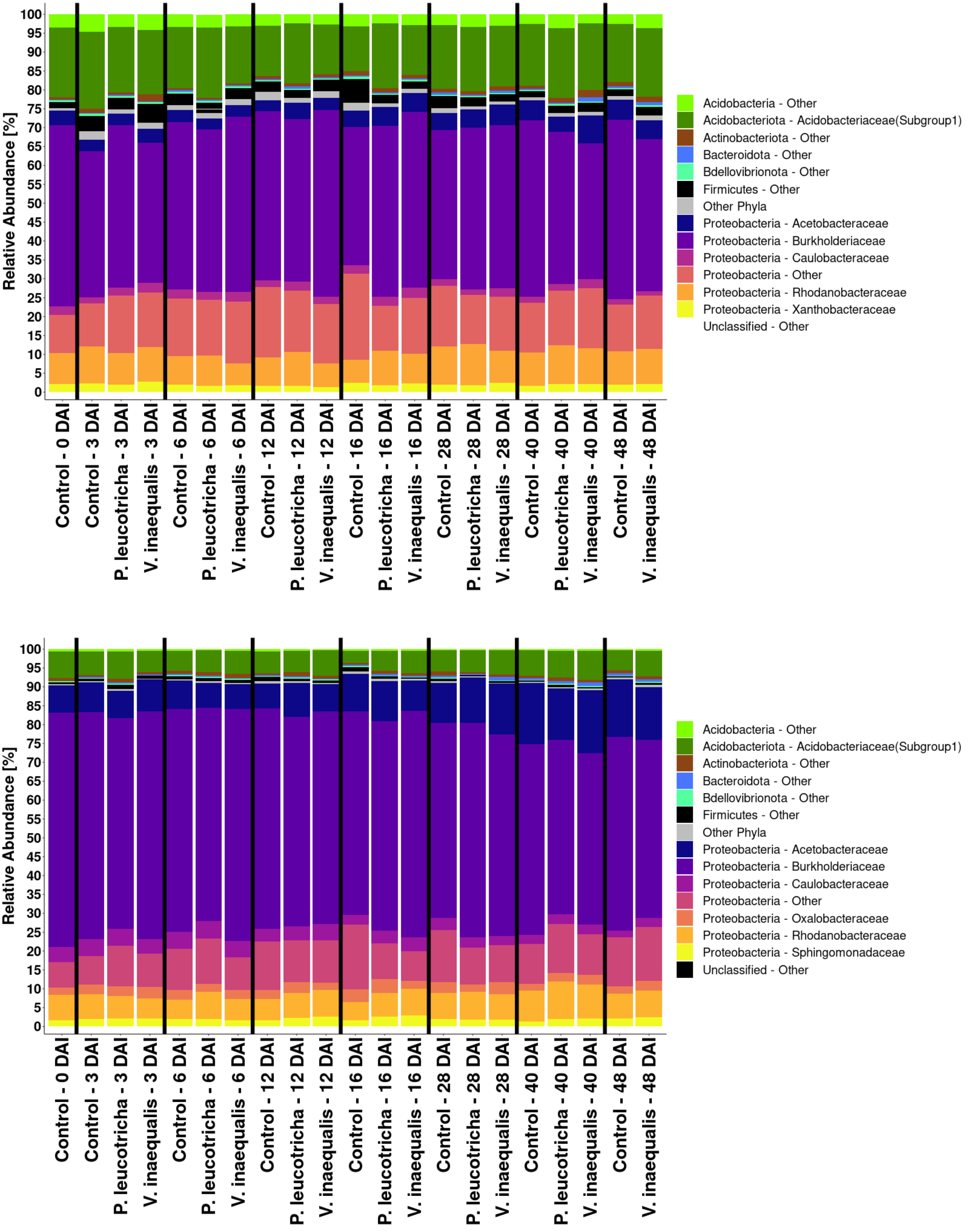

Figure S1. Composition of the root-associated bacterial community of apple plants as revealed by 16S rRNA gene amplicon sequencing in the temporal trial. The relative abundance of bacterial families in samples from three different treatments (*P. leucotricha*, *V. inaequalis* and a negative control) sampled at different days after inoculation (DAI) in the loosely associated (L, upper panel) and tightly associated (T, lower panel) compartment is shown. Phyla and their families with < 2 % relative abundance in the respective treatment were grouped as "Other".

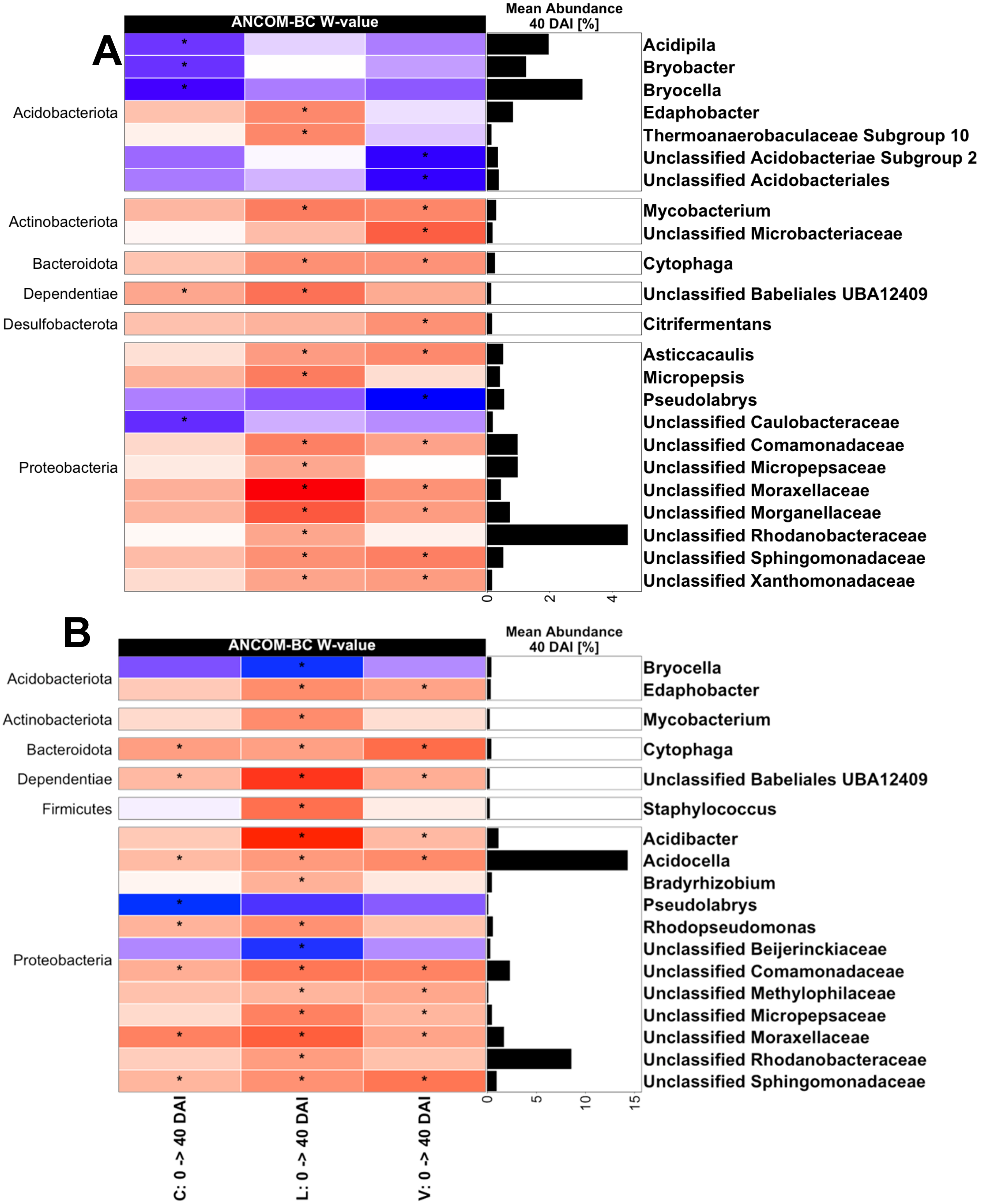

Figure S2. Differential abundance analysis of genera in the L- and T-compartment (panel A and B, respectively) in dependence on pathogen infection 40 days after inoculation (DAI) compared to 0 DAI based on ANCOM-BC. Plants were either inoculated with *V. inaequalis* (V) or *P. leucotricha* (L) and are shown besides an uninoculated control treatment (C). The heatmap shows the coefficients obtained from the ANCOM-BC log-linear model divided by their standard error (called W-value). The colour code indicates differential abundances of genera between the two timepoints with red indicating an increase in relative abundance at 40 DAI compared to 0 DAI. A "*" is shown if ANCOM-BC showed significant differences using the adjusted *p*-value in this comparison. The mean relative abundances of the taxa are displayed at 40 DAI in percent.

In the L-compartment, most identified genera of the pathogen inoculated plants belong to the phylum *Proteobacteria*, e.g., unclassified members of the *Comamonadaceae*, *Moraxellaceae*, *Morganellaceae*, *Sphingomonadaceae* and *Xanthomonadaceae*. In the control plants, several *Acidobacteriota* such as *Acidipila*, *Bryobacter* or *Bryocella* were significantly decreased in relative abundance at 40 DAI, though not in the inoculated plants. Only few observations like these were made in the T-compartment with *Edaphobacter*, *Acidibacter* and unclassified members of *Methylophilaceae* and *Micropepsaceae* being significantly increased in the inoculated plants 40 DAI, but not in the control plants.

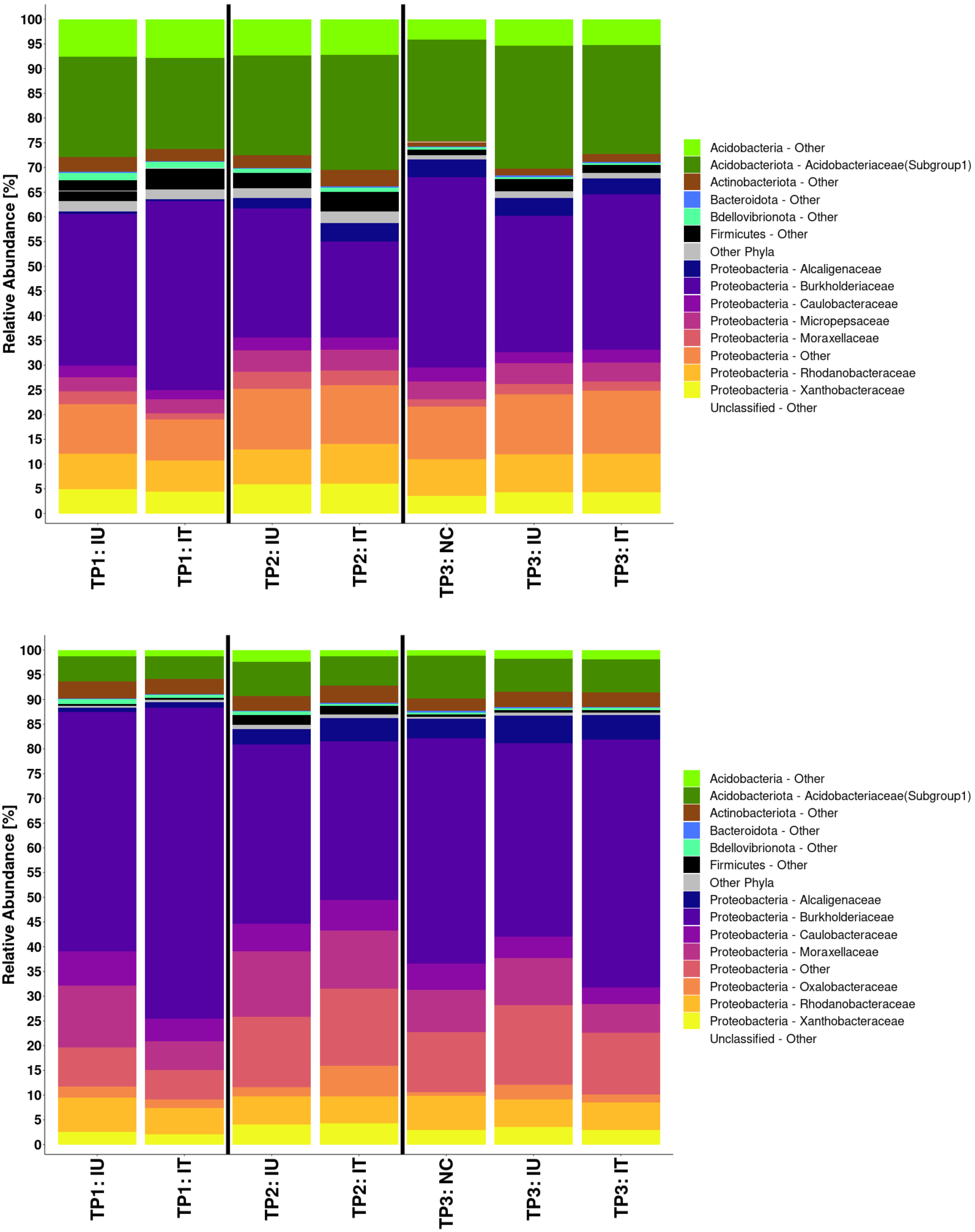

Figure S3. Composition of the root-associated bacterial community of apple plants as revealed by 16S rRNA gene amplicon sequencing in the mixed trial. The relative abundance of bacterial families in samples from three different treatments (IU: inoculated & untreated, IT: inoculated & treated, and NC: negative control) at three different timepoints (TP) in the loosely associated (L, upper panel) and tightly associated (T, lower panel) compartment is shown. Phyla and their families with < 2 % relative abundance in the respective treatment were grouped as "Other".

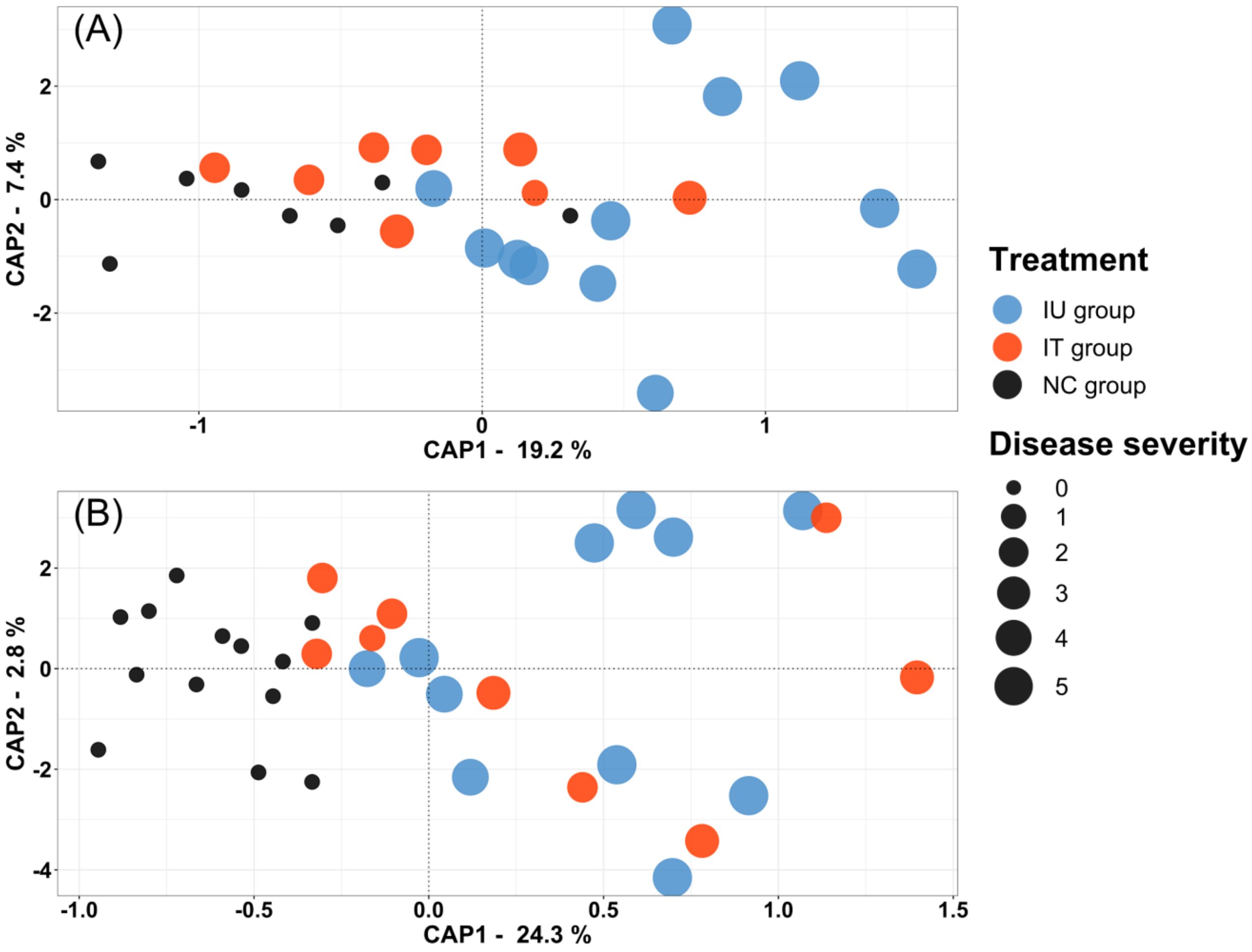

Figure S4. Variation in beta diversity of differently treated apple saplings at timepoint (TP) 3 in the L-compartment (upper panel) and T-compartment (lower panel). Variation is presented based on constrained analysis of principle coordinates (CAP) using DEICODE distance matrices; it is constrained by the variables treatment and disease severity. Plants were either inoculated with *P. leucotricha* and left untreated (IU) or were additionally treated with a synthetic fungicide (IT), or they underwent a treatment with water as control (NC). The different treatments are shown in different colors, and disease severity is illustrated by different symbol sizes, rated on a 0-5 scale with 0 = healthy plants and 5 = plants having multiple leaves entirely covered with mycelium and with leaves close to senescence.

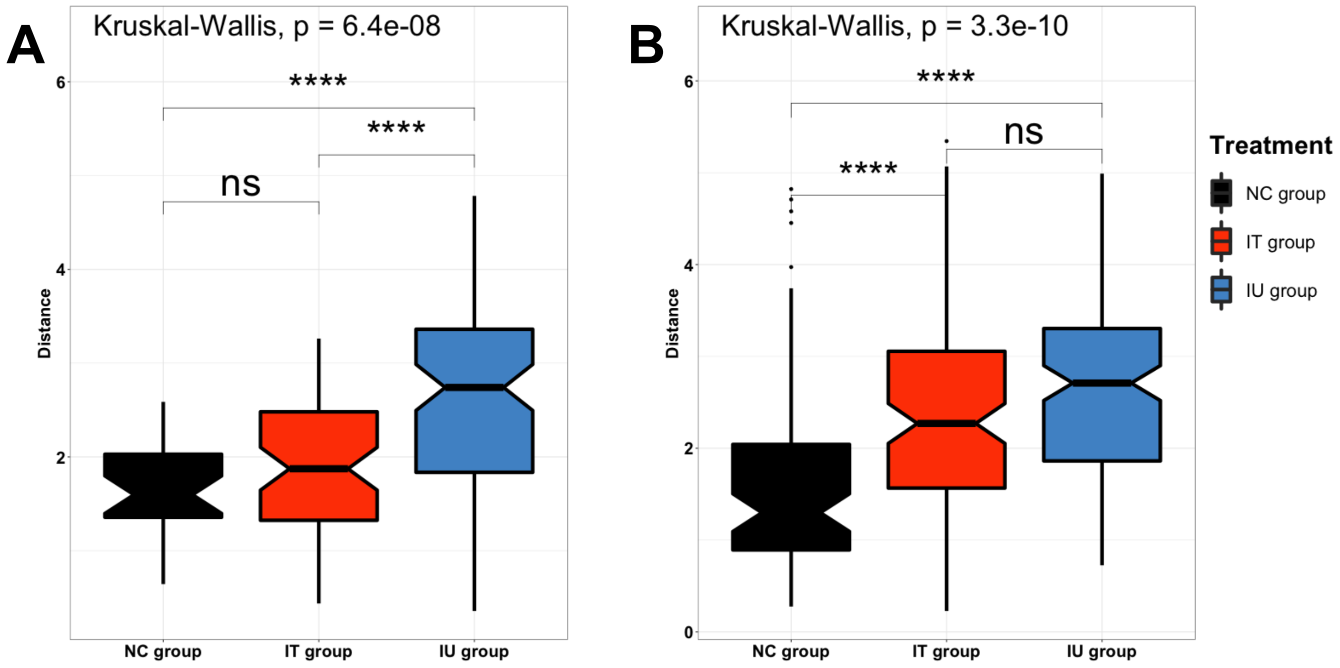

Figure S5. Boxplots showing the differences between two different treatments (IT and IU) to an untreated control group (NC group) for the (A) L-compartment and (B) T-compartment at TP3 based on DEICODE distances. Significant differences were calculated with pairwise Kruskal-Wallis tests ("***"" = *p*-value of < 0.001, ns = non significant).
